# Novel signals of adaptive genetic variation in northwestern Atlantic cod revealed by whole‐genome sequencing

**DOI:** 10.1111/eva.12861

**Published:** 2019-09-13

**Authors:** Gemma V. Clucas, R. Nicolas Lou, Nina O. Therkildsen, Adrienne I. Kovach

**Affiliations:** ^1^ Natural Resources and the Environment University of New Hampshire Durham NH USA; ^2^ Department of Natural Resources Cornell University Ithaca NY USA

**Keywords:** adaptive differentiation, allochronic reproduction, Atlantic cod, chromosomal inversions, fisheries management, heat shock proteins, population genomics, whole‐genome resequencing

## Abstract

Selection can create complex patterns of adaptive differentiation among populations in the wild that may be relevant to management. Atlantic cod in the Northwest Atlantic are at a fraction of their historical abundance and a lack of recovery within the Gulf of Maine has created concern regarding the misalignment of fisheries management structures with biological population structure. To address this and investigate genome‐wide patterns of variation, we used low‐coverage sequencing to perform a region‐wide, whole‐genome analysis of fine‐scale population structure. We sequenced 306 individuals from 20 sampling locations in U.S. and Canadian waters, including the major spawning aggregations in the Gulf of Maine in addition to spawning aggregations from Georges Bank, southern New England, the eastern Scotian Shelf, and St. Pierre Bank. With genotype likelihoods estimated at almost 11 million loci, we found large differences in haplotype frequencies of previously described chromosomal inversions between Canadian and U.S. sampling locations and also among U.S. sampling locations. Our whole‐genome resolution also revealed novel outlier peaks, some of which showed significant genetic differentiation among sampling locations. Comparisons between allochronic winter‐ and spring‐spawning populations revealed highly elevated relative (*F_ST_*) and absolute (*d_xy_*) genetic differentiation near genes involved in reproduction, particularly genes associated with the brain‐pituitary‐gonadal axis, which likely control timing of spawning, contributing to prezygotic isolation. We also found genetic differentiation associated with heat shock proteins and other genes of functional relevance, with complex patterns that may point to multifaceted selection pressures and local adaptation among spawning populations. We provide a high‐resolution picture of U.S. Atlantic cod population structure, revealing greater complexity than is currently recognized in management. Our genome‐scan approach likely underestimates the full suite of adaptive differentiation among sampling locations. Nevertheless, it should inform the revision of stock boundaries to preserve adaptive genetic diversity and evolutionary potential of cod populations.

## 1 INTRODUCTION

Knowledge of patterns and drivers of adaptive genetic variation in natural populations provides insight into ecological and evolutionary processes that influence biodiversity and is crucial for species conservation and predicting organismal responses to environmental change (Bay et al., 2018; Conover, Clarke, Munch, & Wagner, 2006; Li et al., 2018). As genomic tools advance, so does our understanding of the complex patterns of adaptive differentiation found among populations in the wild. Whole‐genome sequencing (Jones et al., 2012; Narum, Di Genova, Micheletti, & Maass, 2018), gene expression analysis (Alvarez, Schrey, & Richards, 2015), and epigenetics (Luyer et al., 2017) have all been used to identify variants linked to local adaptation and to characterize the adaptive capacity of species (Harrisson, Pavlova, Telonis‐Scott, & Sunnucks, 2014; Hoban et al., 2016). In the marine realm, examples of locally adapted populations are abundant, but remain poorly understood, especially in high gene flow systems (Hauser & Carvalho, 2008; Li et al., 2018; Sanford & Kelly, 2011). Large effective population sizes of marine species (weak genetic drift) and high gene flow often create a background of low differentiation among populations (DeWoody & Avise, 2000) against which outlier peaks can be identified with genomic scans for selection (Pespeni & Palumbi, 2013; Pujolar et al., 2014). How these signals of putative adaptation should be interpreted with respect to population structure and in turn considered for management of exploited marine species remains a challenge (Conover et al., 2006; Funk, McKay, Hohenlohe, & Allendorf, 2012; McMahon, Teeling, & Höglund, 2014).

Atlantic cod (*Gadus morhua*) is a high value, commercially exploited species for which questions about adaptive diversity are paramount. Many studies have identified genomic signals of adaptive divergence among cod populations against a background of low genome‐wide differentiation (Barney, Munkholm, Walt, & Palumbi, 2017; Barth et al., 2017; Berg et al., 2015, 2017, 2016; Bradbury et al., 2010, 2013; Hemmer‐Hansen et al., 2013; Kirubakaran et al., 2016; Nielsen et al., 2009; Sinclair‐Waters et al., 2017; Sodeland et al., 2016; Therkildsen, Hemmer‐Hansen, Als, et al., 2013; Therkildsen, Hemmer‐Hansen, Hedeholm, et al., 2013). A wide geographic distribution across a variety of habitats and distinctive life‐history strategies, such as migratory and resident ecotypes, are thought to underlie these diverse signals of selection. However, a history of over‐exploitation and stock collapse may have already caused some of this diversity to be lost (Ames, 2004), while putting what is left at risk. Recognizing and preserving adaptive diversity in management structures is important for maintaining resilience and recovery of stocks (Hilborn, Quinn, Schindler, & Rogers, 2003; Kerr, Cadrin, & Secor, 2010b) through portfolio effects (Schindler et al., 2010), particularly in an era of rapid environmental change, to which cod are known to be sensitive (Drinkwater, 2005; Pershing et al., 2015).

The majority of research into the adaptive evolution of cod has focused on large chromosomal inversions that are found across four linkage groups (LG 1, 2, 7, and 12) and, in total, account for around 7% of the genome (Barth et al., 2017; Berg et al., 2017, 2016; Kirubakaran et al., 2016; Sodeland et al., 2016). The inversions have been variously linked to resident/migratory and inshore/offshore ecotypes (LG 1, 2, 7, and 12; Berg et al., 2016, 2017; Hemmer‐Hansen et al., 2013; Kess et al., 2018; Kirubakaran et al., 2016; Sinclair‐Waters et al., 2017, 2018; Therkildsen, Hemmer‐Hansen, Hedeholm, et al., 2013), thermal adaptation (LG 1, 2, 7 and 12; Barney et al., 2017; Berg et al., 2017; ; Bradbury et al., 2010, 2013, 2014; Therkildsen, Hemmer‐Hansen, Als, et al., 2013; Therkildsen, Hemmer‐Hansen, Hedeholm, et al., 2013), salinity (LG 1 and 2; Barth et al., 2017; Berg et al., 2015), and oxygen concentrations (LG 1, 2, and 7; Berg et al., 2015). Much less is known about variation in regions of the genome outside of these chromosomal inversions, in part because almost all prior studies have used SNP arrays or reduced representation techniques with relatively low marker density genome‐wide.

**Figure 1:**
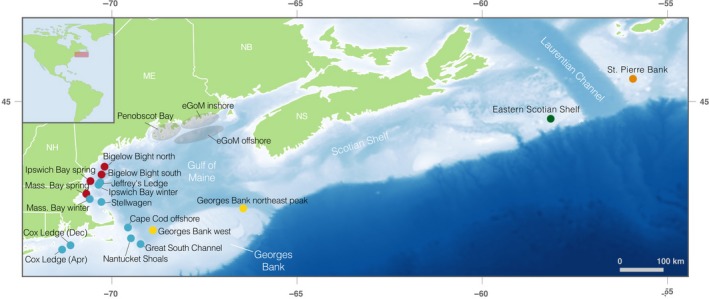
A map of the sampling locations used in the study. Locations of spawning aggregations sampled are shown by the colored points. St. Pierre Bank and the eastern Scotian Shelf are Canadian sampling locations; all other samples come from U.S. or transboundary waters. The colors represent our a priori understanding and expectations of the population structure: red and blue = northern spring coastal complex and southern complex, respectively, of Kovach et al. (2010); yellow = Georges Bank; green = eastern Scotian Shelf; orange = St. Pierre Bank. The ranges within which the nonspawning fish from the eGoM were caught are shown by the gray circles

In the Northwest (NW) Atlantic, variation in the frequency of the chromosomal inversions has been found to underlie much of the population structure at both large (Berg et al., 2017; Bradbury et al., 2010, 2013) and small spatial scales (Barney et al., 2017; Clucas, Kerr, et al., 2019; Sinclair‐Waters et al., 2018). Within the Gulf of Maine (GoM), additional outliers outside of the inversions have also been noted (Clucas, Kerr, et al., 2019) while previous studies primarily using microsatellite markers similarly pointed to the role of adaptive differentiation in driving population structure in this region of the NW Atlantic (Kovach, Breton, Berlinsky, Maceda, & Wirgin, 2010). However, the *Pan I* and *Gmo132* loci that were found to be under selection in Kovach et al., (2010) can be localized to the inversions on LG 1 (Kirubakaran et al., 2016) and LG 7 (this study), respectively, highlighting the roles of these inversions in driving population structure. Perhaps the most interesting pattern uncovered by these earlier studies is the existence of allochronic populations (i.e., population segments that spawn in different seasons), with separate winter‐spawning (December–March) and spring‐spawning (May–June) groups within two bays—Ipswich Bay and Massachusetts Bay—in the western GoM (wGoM; Figure 1) that are genetically distinct (Kovach et al., 2010; Wirgin et al., 2007) and differ in haplotype frequencies at the inversions on LG 2, 7, and 12 (Barney et al., 2017; Clucas, Kerr, et al., 2019). These allochronic populations may have different thermal adaptations associated with timing of spawning or juvenile settlement (Barney et al., 2017; Kovach et al., 2010) or adaptations associated with adult movements to different depths and salinities (Kovach et al., 2010). These findings, combined with a recent effort to re‐evaluate stock structure of cod in U.S. waters (Annala, 2012), have generated great interest in a comprehensive population genomic study of cod in this region to better understand the population structure and distribution of adaptive genetic variation with the unprecedented resolution afforded by recent advances in population‐scale whole‐genome resequencing.

Here, we use low‐coverage whole‐genome resequencing of 306 individuals from 20 sampling locations throughout the U.S. and adjacent Canadian waters (Figure 1) to (a) characterize the population structure of cod in this area, using genome‐wide markers, and compare the U.S. sampling locations to nearby Canadian locations and (b) investigate patterns of differentiation across the whole genome, including at regions outside of the previously studied chromosomal inversions, particularly between allochronic populations and populations at the northern and southern boundaries of our sampling range that experience different thermal regimes. We evaluate our findings with respect to current cod stock boundaries to inform decision‐making about stock management and assessment.

## 2 METHODS

### 2.1 Study area and sample collection

The study area includes sampling locations from all known, primary cod spawning grounds in U.S. waters, including those in eastern and western GoM (eGoM and wGoM, respectively), the northeastern peak and western flank of Georges Bank, the Great South Channel area, southern New England waters off Cox Ledge, and samples from Canadian waters on the eastern Scotian Shelf (management unit 4VsW) and St. Pierre Bank off Newfoundland (management unit 3Ps; totaling 20 sampling sites, Figure 1). Prior studies have suggested that cod spawning in U.S. waters are genetically differentiated into three spawning complexes (Kovach et al., 2010): (a) the northern spring coastal complex (red points on Figure 1) consisting of spring‐spawning aggregations in the inshore wGoM; (b) the southern complex (blue points on Figure 1) consisting mainly of fall and winter spawners from the inshore and nearshore wGoM, Great South Channel area, and Cox Ledge; and (c) cod spawning on the northeastern peak of Georges Bank (Kovach et al., 2010). We considered these three a priori groupings and we hypothesized that western Georges Bank spawners would be more closely related to the northeastern peak (yellow points) than other sampling locations as a result of the clockwise gyre on the bank (Lough et al., 2006) and the depth of the Great South Channel, which would serve to separate the populations on Georges Bank from those to the west of the Channel. The relationship of fish caught in eGoM to other spawning groups is still uncertain (Clucas, Kerr, et al., 2019) due to the current lack of active spawning aggregations in that area. It is also unknown how the Canadian sampling locations in this study relate to the Gulf of Maine sampling locations, although Berg et al., (2017) showed that Canadian cod from north of the Laurentian Channel were differentiated from offshore GoM cod. We hypothesized that the Canadian samples would be genetically distinct from one another given the depth of the Laurentian Channel that separates them (green and orange points on Figure 1).

We sequenced 12–15 individuals from each sampling location (see Table S1 for full details). With the help of local fishermen, fishing efforts were specifically targeted toward active spawning aggregations on well‐known spawning grounds (Figure 1). Fin clips were taken from individuals that were either in spawning condition or recently spawned to minimize the effects of including nonspawning migrants on our estimates of population structure, with the exception of the samples from Canada, for which we did not have information about individual reproductive status (although they were sampled during the spawning season). Due to the lack of active spawning in the eGoM, fin clips from this area were collected from nonspawning cod caught by the Maine Center for Coastal Fisheries' Sentinel Hook Survey (Henry, 2013; Rodrigue, 2017) from stations located throughout mid‐coast and downeast Maine. Exact locations for some of these individuals were unavailable, so we present outlines of the areas in which they were caught in Figure 1. Note that in Ipswich and Massachusetts Bays, two collections were made during the two distinct spawning seasons—winter (December/January) and late spring (May/June). Cox Ledge samples collected in December and April were both representatives of winter spawners since the spring‐spawning season does not begin until May.

### 2.2 DNA extraction, library preparation, and sequencing

DNA was extracted from fin clip samples with Qiagen® DNeasy Tissue Kits (Qiagen) or with Omega EZNA Tissue DNA kits (Omega Bio‐Tek), following the manufacturer's protocols. We prepared a separate, dual indexed library for each individual (total *n* = 333) with a highly cost‐effective protocol based on Illumina's Nextera reagents, as described in Therkildsen and Palumbi (2017). The libraries were sequenced across a total of 6 lanes of paired‐end 125‐bp reads on an Illumina HiSeq 2500 (v4 chemistry) at the University of Utah's Bioinformatics Core Facility. To even out the data yield among samples, we sequenced in two batches with the second batch adding more sequences to the 130 libraries that had initially obtained the lowest read output.

### 2.3 Sequence filtering, alignment, and genotype likelihood estimation

We used FastQC v0.11.5 (Andrews, 2010) to check read quality and Trimmomatic v0.36 (Bolger, Lohse, & Usadel, 2014) to remove adapters using the ILLUMINACLIP mode allowing two mismatches, with a palindrome clip threshold of 30, a simple clip threshold of 10, a minimum adapter length of four, and keeping both reads after clipping. We aligned reads to the Atlantic cod gadMor2 reference genome (Tørresen et al., 2017) using Bowtie 2 v2.2.8 (Langmead & Salzberg, 2012) in end‐to‐end mode with the following settings: the “very‐sensitive” preset option, a minimum fragment length of zero, and a maximum fragment length of 1,500. We filtered mapped reads using samtools v1.8 (http://www.htslib.org/) to remove nonuniquely mapped reads and reads with a mapping quality score less than 20. We removed duplicate reads using the Picard v2.9.0 MarkDuplicates tool (http://broadinstitute.github.io/picard/). We then merged the bam files from individuals that had been sequenced twice using samtools, deduplicated again, and clipped redundant sequence from overlapping ends of each mapped read pair using the bamutil v1.0.14 clipOverlap tool (Jun, Wing, Abecasis, & Kang, 2015) with default settings. Finally, we realigned reads around indels using the GATK v.3.7 IndelRealigner tool (McKenna et al., 2010), creating target intervals across all individuals and using the default settings for realignment. We calculated coverage statistics using the samtools depth tool.

We used ANGSD v0.912 (Korneliussen, Albrechtsen, & Nielsen, 2014) to call SNPs and estimate genotype likelihoods using the samtools model (‐GL 1). SNPs were called with this data set, 172 historical samples (analyzed in a separate study but called jointly so that direct comparisons can be made in future work between modern and historical datasets), and 15 additional lab‐reared samples from the west coast of Nova Scotia that were later dropped due to a high number of half‐siblings among those individuals. Thus, SNPs were called across a total of 505 individuals. We set the minimum number of individuals' threshold to 100 (i.e., considering only genomic locations with data from at least 100 individuals, ‐minInd 100). We excluded bases with a base quality score <20 (‐minQ 20) and applied a maximum total depth threshold of 1,000 to remove loci from repetitive regions (‐setMaxDepth 1000). This threshold was chosen since our target sequencing depth was 1X, and it was deemed unlikely that each individual in the data set would have received approximately 2X coverage at a given site if it were not from a repetitive region (see Figure S1 for the depth distribution of the retained loci). We used a *p*‐value cutoff of 10^–6^ for calling polymorphic loci (‐SNP_pval 1e‐6) and retained only SNPs with a minor global allele frequency ≥1% (‐minMaf 0.01). Major and minor alleles were inferred from genotype likelihoods across all individuals (‐doMajorMinor 1), and these were then set for downstream analyses (‐doMajorMinor 3). Per‐individual inbreeding coefficients were estimated using PCAngsd v0.93 (Meisner & Albrechtsen, 2018) with the simple estimator (‐inbreed 2).

At this stage, we removed four individuals from our data set that had missing data for almost 100% of SNPs. We also removed a collection of samples from inshore waters off Cape Cod, as we likely had too few individuals from this site (total of 8) to accurately estimate minor allele frequencies, leading to very low expected heterozygosity for this sampling location. All further analyses were conducted with the 306 remaining individuals from 20 sampling locations, all of which included 11–15 individuals each, except for Stellwagen and the northeastern peak of Georges Bank, which included 23 and 25 individuals, respectively, after we combined two sets of samples from each location (Table S1).

### 2.4 Population genomics

Weighted pairwise *F_ST_* was estimated between each sampling location using ANGSD. First, we estimated site allele frequency likelihood for each sampling location (‐doSaf 1), supplying the reference genome and setting the minimum number of individuals threshold (‐minInd) to either (a) three or, (b) two‐thirds of the number of individuals in the sampling location to test the effect of missing data on pairwise *F_ST_* estimates. We then estimated the 2D site frequency spectrum for each pair of populations (realSFS) and calculated the average pairwise weighted *F_ST_* (realSFS fst). Classical multidimensional scaling (MDS) was applied to the pairwise *F_ST_* matrix using the cmdscale function in R (R Core Team, 2014) to visualize the genetic differentiation among all sampling locations. We also investigated whether small and unequal sample sizes were likely to limit our ability to determine population structure by randomly down‐sampling to include only eight individuals per sampling location. We recalculated pairwise *F_ST_*, setting the minimum number of individuals' threshold to three, as before, and applied MDS to the pairwise *F_ST_* matrix.

To explore genetic variation within sampling locations, we used PCAngsd (Meisner & Albrechtsen, 2018) to calculate a covariance matrix among individuals and then performed an individual‐level principal component analysis (PCA) using the eigen function in R, using default settings. It was impossible to view individuals from all 20 sampling locations on the same plot due to overlapping points, so we plotted each sampling location separately, although the PCA was performed across all individuals in all sampling locations.

To investigate the influence of the genomic inversions on LG 1, 2, 7, and 12, we also used PCAngsd to perform PCAs separately for each genomic region containing an inversion. Within inversion regions, individuals formed three clusters along PC1 and we used the position of individuals along this axis to genotype them as homozygous for the noninverted haplotype, heterozygous, or homozygous for the inverted haplotype (Ma & Amos, 2012). We could not determine the ancestral orientation of each inversion, so we arbitrarily defined these clusters as AA, AB, and BB regardless of the ancestral state. With these individual haplotype classifications, we could then calculate haplotype frequencies at each sampling location for each inversion. We performed PCA on the haplotype frequency matrix using the prcomp function from the stats package in R and the factoextra package to visualize the contribution of each inversion to the population structure. We tested for inter‐chromosomal linkage disequilibrium (LD) among the inversions using the LD function from the genetics package in R. We calculated LD separately for each of the groups outlined below, to avoid influences of population structure on LD calculations.

Next, to evaluate genomic patterns of genetic differentiation, we created Manhattan plots of pairwise *F_ST_* in nonoverlapping 15 kb windows using ANGSD (realSFS fst stats2). To reduce the number of pairwise comparisons to investigate, we grouped certain sampling locations based on our a priori knowledge of population structure (Clucas, Kerr, et al., 2019; Kovach et al., 2010; Wirgin et al., 2007), findings of low differentiation among certain sampling locations in the current study, and by groups of interest from a fisheries management perspective. The similarity between the Georges Bank west and northeast peak sampling locations (see results section) led us to group those two locations together. We also created a wGoM spring‐spawning group and a wGoM winter‐spawning group, consisting of winter and spring spawners from Ipswich Bay and Massachusetts Bay, to investigate the genomic basis of differentiation between these allochronic, genetically differentiated spawning groups (Clucas, Kerr, et al., 2019; Kovach et al., 2010). We excluded other samples from the wGoM due to higher variability within these locations in the individual‐level PCA (see results), allowing us to focus on the clearest signal of genomic differentiation associated with spawning timing, and because spawning aggregations in these two bays comprise the majority of spawning activity in wGoM today. Other groups of interest were the Great South Channel sampling locations, consisting of Great South Channel, Nantucket Shoals, and Cape Cod Offshore, the Cox Ledge sampling locations, and the eGoM sampling locations. These three groups have all been hypothesized to be distinct from other spawning groups within the Gulf of Maine or Georges Bank (Ames, 2004; Wise, 1963; Zemeckis, Martins, Kerr, & Cadrin, 2014), and so we investigated them separately. Therefore, we evaluated genome‐wide patterns of differentiation among the following sampling locations and groups: (a) St. Pierre Bank; (b) eastern Scotian Shelf; (c) wGoM spring spawners [Ipswich Bay spring + Massachusetts Bay spring]; (d) wGoM winter spawners [Ipswich Bay winter + Massachusetts Bay winter]; (e) Great South Channel group [Nantucket Shoals + Cape Cod offshore + Great South Channel]; (f) Georges Bank [Georges Bank northeastern peak + Georges Bank west]; (g) Cox Ledge [Cox Ledge (Dec) + Cox Ledge (Apr)]; and (h) eGoM [Penobscot Bay + eGoM inshore + eGoM offshore].

To evaluate putatively neutral patterns of population structure among these groups, we created a putatively neutral SNP data set (hereafter “neutral SNP data set”). We first removed all SNPs within the boundaries of the well‐known chromosomal inversions, calculating the boundaries using ngsLD (Fox, Wright, Fumagalli, & Vieira, 2019) and removing a further 1 Mb either side to account for any uncertainty in estimating the boundaries. We then identified the upper 5% of 15 kb windows in each of the pairwise *F_ST_* comparisons and removed all SNPs within these windows (i.e., we removed the upper 5% of windows in all 28 pairwise comparisons). This effectively removed all SNPs in any *F_ST_* outlier window found between any pair of groups, leaving only putatively neutral regions of the genome. We then estimated the weighted average pairwise *F_ST_* values among groups using the same methods in ANGSD described previously and visualized the result using MDS, as before. In addition, to evaluate putatively neutral patterns of population structure among sampling locations rather than among groups, we subsetted the stringent SNP data set for the neutral SNPs identified above and calculated pairwise *F_ST_* among all sampling locations using the neutral, stringent SNP data set. Finally, we performed an individual‐level PCA using the covariance matrix calculated from the neutral SNP dataset in PCAngsd, as described above.

To investigate regions of elevated differentiation outside of the chromosomal inversions, we chose to focus on five of the 28 pairwise comparisons among groups, which were (a) wGoM spring versus wGoM winter spawners to investigate the genomic basis of alternative spawning times; (b) St. Pierre Bank versus Cox Ledge as these are at the extremes of the geographic and thermal distribution of our samples; (c) Georges Bank versus Cox Ledge as these are currently in the same management unit but pairwise *F_ST_* suggested they are genetically divergent; (d) winter spawners versus eGoM as pairwise *F_ST_* suggested they were surprisingly similar; and (e) spring spawners versus the Great South Channel group as the Manhattan plot between these showed some relatively high peaks of differentiation. We identified the genomic windows that were in the upper 99.9th percentile of the windowed *F_ST_* distribution for each comparison, after excluding the inversions, and extracted all gene annotations from the gadMor2 reference genome (using the filtered gene set, which includes only putatively reliable annotations (Tørresen et al., 2017)) that were within 15 kb of the center of each window, thus investigating a 30 kb window in total. We used UniprotKB (www.uniprot.org) to investigate the function of each gene. For those peaks that appeared to have functional relevance or particularly elevated peaks compared to the genomic background, we estimated pairwise *F_ST_* and Tajima's *D* (Korneliussen, Moltke, Albrechtsen, & Nielsen, 2013) in each group in nonoverlapping 5 kb windows. To investigate patterns of absolute genetic differentiation in these peaks of interest, we also calculated *d_xy_* on a per‐SNP basis using the calcDxy.R script of Joshua Penalba (https://github.com/mfumagalli/ngsPopGen/blob/master/scripts/calcDxy.R).

Finally, we investigated how each of the identified peaks was driving genetic differentiation among all sampling locations. To do so, we first selected the SNPs that underlaid each peak. For inversions, we included all SNPs inside the inverted regions. For each outlier region outside of the inversions, we located all SNPs within the region, and among those SNPs, we selected those with the 1% highest *F_ST_* values in the population pairwise comparison where the peak appeared the most significant (Supp. File 1). Then, based on genotype likelihood data at these SNPs for all sampling locations, we performed PCA at each of the outlier regions using with the software PCAngsd and recorded the average score along PC1 for each sampling location (so that sampling locations closer to each other along PC1 will have more similar scores). We rescaled the average PC1 score so that the populations with the maximum and minimum scores were assigned 1 and −1, respectively, and visualized this rescaled PC1 score on a heat map.

## 3 RESULTS

The 306 individuals that were included in the final data set received on average, 7.7 million reads each (median = 7.0 million, range = 1.8–24 million reads). After adapter trimming, 92% of raw bases were retained. On average, 58% of the raw reads mapped uniquely to the gadMor2 reference genome. Our average duplication rate was 1.9% (median = 1.4%, range = 0.4%–15.2%), and after deduplication and clipping of overlapping read ends, we retained 45% of our raw bases for downstream analysis. The average individual coverage was 0.67X when calculated across the entire reference genome, including regions that we could not map to (median = 0.60, range = 0.16–1.93). Mean depths and inbreeding coefficients across sampling locations were relatively consistent (Figures S2 and S3) and do not appear to correlate with the inferred population structure. Variant calling identified 10,886,831 SNPs when a minimum of three individuals per sampling location were required to have data, hereafter the “full SNP data set,” and 48,220 SNPs when two‐thirds of individuals in each sampling location had data, hereafter the “stringent SNP data set.” The full SNP data set had an average depth, summing across individuals, of 265.4X (Figure S1) corresponding to an average of 0.88X per individual. The stringent SNP data set had an average depth of 610.8X when summing across individuals (Figure S4), corresponding to an average of 2.0X per individual.

**Figure 2:**
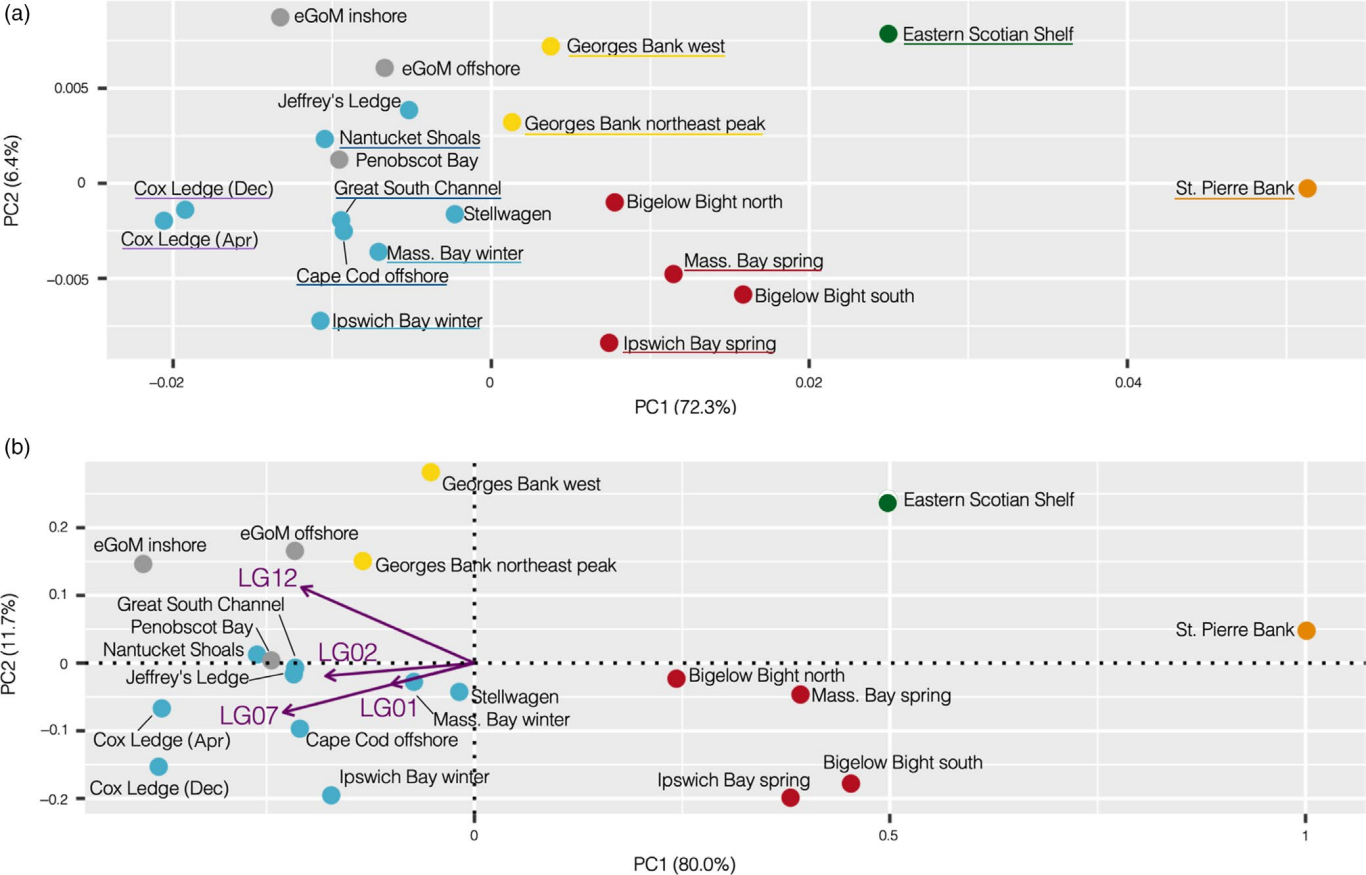
(a) MDS plot showing the population structure based on the pairwise *F_ST_* matrix calculated with the full SNP data set. (b) PCA biplot showing the population structure based solely on haplotype frequencies at the four chromosomal inversions, with the contributions of each of the inversions shown by the purple arrows. The colors represent our a priori understanding and expectations of the population structure: red and blue = northern spring coastal complex and southern complex, respectively, of Kovach et al. (2010); yellow = Georges Bank; green = eastern Scotian Shelf; orange = St. Pierre Bank; gray = eastern GoM. Locations that were grouped for estimating pairwise *F_ST_* among groups are underlined in the same colors

Across the 20 sampling locations, MDS of the weighted pairwise *F_ST_* matrix calculated from genotype likelihoods for the full SNP data set allowed us to visualize the population structure (Figure 2a). Sampling locations were predominantly distinguished by their position along PC1, which explained 72.3% of the variation. The population structure largely followed our a priori expectations, with some exceptions. The Canadian samples from St. Pierre Bank and the eastern Scotian Shelf were divergent from the U.S. samples and from one another, as expected, based on their northerly locations and the deep Laurentian Channel that separates them. Within U.S. waters, the northern spring coastal complex (red points) clustered away from the southern complex (blue points), in agreement with expectations (Kovach et al., 2010; Wirgin et al., 2007). Winter and spring spawners collected from the same bays (Ipswich Bay and Massachusetts Bay) thus clustered by their spawning season rather than geographic location. The Georges Bank samples (yellow) clustered together and were intermediate between northern and southern complexes, also as expected (Kovach et al., 2010). However, the Cox Ledge samples did not cluster with the rest of the southern complex but were instead highly separated along PC1. The eGoM samples (gray points) overlapped with the wGoM and Great South Channel samples of the southern complex on PC1. Penobscot Bay also clustered with the southern complex on PC2, while the more northern eGoM inshore and offshore samples were slightly divergent from that group on PC2. Almost identical patterns of differentiation were found when we repeated these analyses with the stringent SNP data set (Figure S5a) and when we down‐sampled to eight individuals per sampling location (Figure S5b), suggesting that missing data and small sample sizes did not affect our ability to infer population structure. We present the pairwise *F_ST_* matrix calculated with the stringent SNP data set in Table S2.

Individual‐level PCA (Figure S6) confirmed the results from the population level MDS while illuminating within‐location variation. For the most part, variation within sampling locations was low; most individuals clustered together into two or three clusters per sampling location. These clusters likely represent individuals that are homozygous and heterozygous for the chromosomal inversions, creating characteristic clustering patterns (Lotterhos, 2019). This, and the overlap of points across sampling locations, highlights that the chromosomal inversions are mainly driving population structure, but that differences are not fixed. Three sampling locations – Eastern Scotian Shelf, Bigelow Bight south, and Georges Bank west – showed higher within‐location variation with individuals spread among multiple clusters. Individuals from the Eastern Scotian Shelf represent collections from multiple sites on the Eastern Scotian Shelf, which could explain their higher variability within this group, while the inclusion of spent individuals in Bigelow Bight south may also explain the higher variability at this location; individuals could have spawned elsewhere before moving into the sampling area, thus not representing the true, local spawning aggregation.

Further evidence that haplotype frequencies of the chromosomal inversions (Figure S7) were driving much of the population structure can be seen in Figure 2b, which shows the population structure captured by just the inversions and is highly similar to the structure inferred from the full SNP data set (Figure 2a). The PCA biplot shows that the inversions on LG 1, 2, and 7 all contributed in a similar fashion, strongly differentiating the Canadian samples from the southern complex and Georges Bank, while the northern spring complex was intermediate between these. LG 7 had the greatest effect out of these three inversions. The inversion on LG 12 had a slightly different effect (Figure 2b). We did not find evidence for the inversions to be in inter‐chromosomal linkage disequilibrium as all correlation coefficients were close to zero (Tables S3–S10). See Table S11 for the estimated boundaries of the inversions.

**Figure 3:**
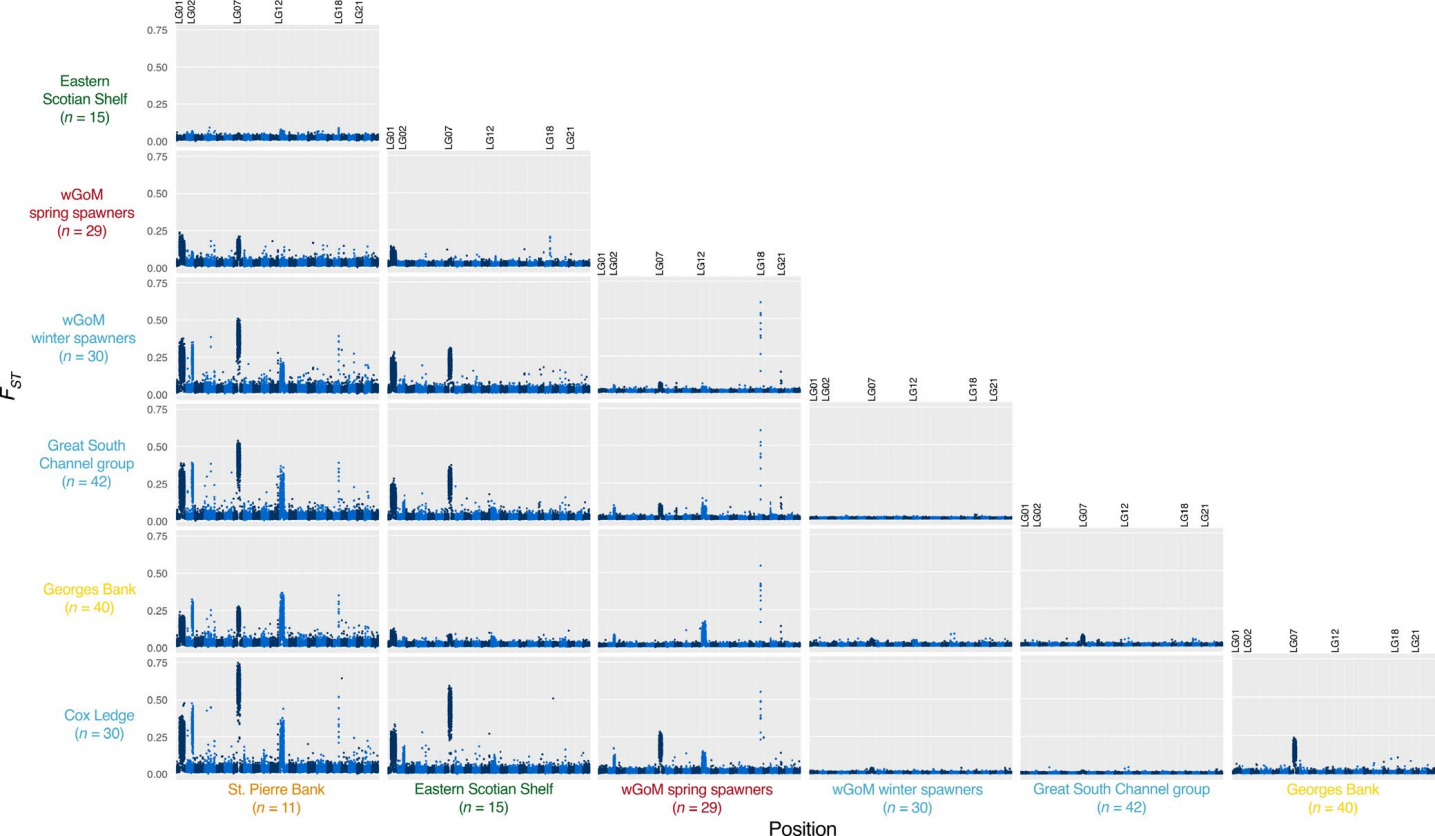
Manhattan plots displaying pairwise *F_ST_* estimated in 15 kb windows among sampling locations and groups. Within each pairwise comparison, LG 1 to LG 23 are displayed from left to right with alternating dark blue and light blue colors. The positions of LGs with significant peaks discussed in the text are labeled. The colors of the labels represent our a priori understanding and expectations of population structure, as in Figure 1. The number of individuals in each group is given in parentheses

The significant contributions of these inversions to the population structure and the low levels of background differentiation among sampling locations and groups could clearly be seen in the Manhattan plots displaying pairwise *F_ST_* in 15 kb windows (Figure 3). LG 1 appeared to differentiate the Canadian samples (St. Pierre Bank and eastern Scotian Shelf) from all other groups. LG 7 also differentiated the Canadian samples from U.S. samples, except wGoM spring spawners, and there were noticeable peaks at LG 7 between wGoM spring spawners and other U.S. groups, and between Cox Ledge and Georges Bank. LG 2 differentiated the Canadian samples and wGoM spring spawners from all other groups. LG12 showed a similar pattern, although peaks were absent in comparisons involving the eastern Scotian Shelf and wGoM spring and winter spawners. Larger versions of these plots, including plots involving the eGoM nonspawning samples, are available in the Figures S8–S15. The eGoM nonspawning samples showed very little differentiation from the wGoM winter spawners, the Great South Channel group, Georges Bank, and Cox Ledge (Figure S15).

Outside the *F_ST_* peaks created by the chromosomal inversions, there were other noticeable peaks of elevated divergence on LGs 4, 8, 9, 11, 18, 20, and 21, which we investigated in the following comparisons: (a) wGoM spring spawners versus wGoM winter spawners; (b) St. Pierre Bank versus Cox Ledge; (c) Georges Bank versus Cox Ledge; (d) winter spawners versus eGoM; and (e) spring spawners versus Great South Channel group (Figure S16). A table of the genes associated with the outlier peaks in all five of these comparisons can be found online (Supp. File 1). The most significant of these peaks (those that occurred in more than one of the pairwise comparisons or that involved multiple adjacent outlier windows) and their associated gene annotations are shown in Table 1. Four out of eight of these peaks involved genes with known reproductive functions (genes HSD17B2, FSHR, NME8, and ESR2) while a fifth peak overlapped two heat shock protein‐coding genes (HSPB1, HSPB8). This *F_ST_* peak was most highly elevated in the comparison between Cox Ledge and St. Pierre Bank (Figure S17), which were at the northern and southern extremes of our sampling range. However, *d_xy_* was not elevated in this region (Figure S17).

**Table 1:** Significant outlier peaks and the genes annotated within these regions

LG	Region (Mb)	Gene name	Description
4	31.99–32.51	NLRC3	Regulator of the innate immune response
		CNOT4	Involved in the protein ubiquitination pathway
8	2.32–2.33	NLRC3	Regulator of the innate immune response
		HSPB1	Small heat shock protein
		HSPB8	Displays temperature‐dependent chaperone activity (heat shock protein beta‐8)
9	6.45–6.49	GPI	Glycolytic enzyme involved in glycolysis
		HSD17B2	Catalyses the interconversion of testosterone and androstenedione, as well as estradiol and estrone
		MPHOSPH6	RNA‐binding protein that associates with the RNA exosome complex
		DBX1	A homeobox protein
11	26.40–26.49	MUC2	Coats the epithelia of mucus membranes
18	17.06–17.19	FSHR	G protein‐coupled receptor for follitropin, the follicle‐stimulating hormone
		RASD1	Small GTPase
		PEMT	Involved in phosphatidylcholine biosynthesis
20	0.05–0.65	SMARCAL1	Re‐winds stably unwound DNA
		NME8	Involved in spermatogenesis
		DNAJC10	Involved in protein folding in the endoplasmic reticulum
		NUP35	Functions as a component of the nuclear pore complex
21	8.18–8.22	ESR2	An estrogen receptor
21	10.17–10.20	SYNE2	Has a role in maintaining subcellular spatial organization

**Figure 4:**
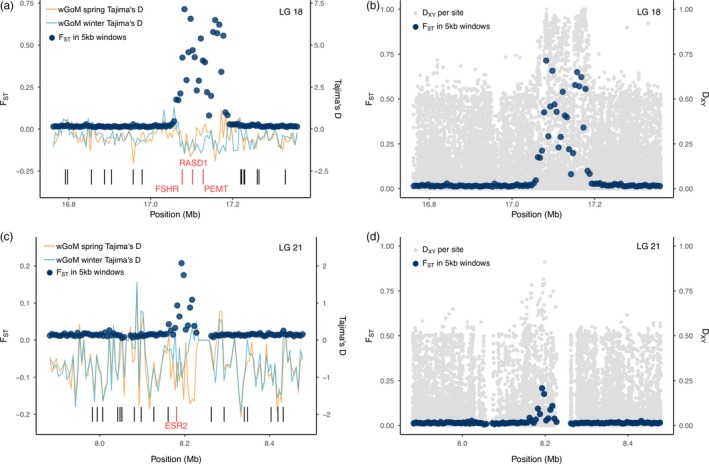
(a) Pairwise *F_ST_* estimated in 5 kb windows for the outlier peak on LG 18 between wGoM spring spawners and wGoM winter spawners (dark blue points). Tajima's *D*, estimated in 5 kb windows, is displayed for each group by the orange (spring spawners) and light blue lines (winter spawners). The locations of gene annotations are shown by the black bars, with the red bars highlighting the genes under the peak. (b) Pairwise *F_ST_*, estimated in 5 kb windows, is overlaid over per‐SNP estimates of *d_xy_* between spring and winter spawners for the same region of LG 18 as in part a. (c) Pairwise *F_ST_* and Tajima's *D*, estimated in 5 kb windows, for the outlier peak on LG 21, also for the wGoM spring spawners and wGoM winter spawners. Colors are the same as in a. (d) Pairwise *F_ST_*, estimated in 5 kb windows, is overlaid over per‐SNP estimates of *d_xy_* for the same region of LG 21 as in part c

The most pronounced of all the outlier peaks occurred on LG 18 and was found in comparisons between the wGoM spring spawners and other groups within U.S. waters that spawn in the winter, as well as between St. Pierre Bank and all U.S. groups except the spring spawners (Figure 3). This peak reached a maximum average *F_ST_* of 0.71 (calculated in 5 kb windows) between wGoM winter and wGoM spring spawners and overlies three genes: FSHR, RASD1, and PEMT (Figure 4). FSHR codes for a follicle‐stimulating hormone receptor. More negative Tajima's *D* was recorded under the *F_ST_* peak in wGoM winter spawners compared to spring spawners, although both populations deviated from zero and Tajima's *D* patterns were complex (Figure 4a). There was a concomitant increase in *d_xy_* with *F_ST_* in this region (Figure 4b). Two further peaks with highly elevated *F_ST_* were found on LG21 in comparisons between wGoM spring‐ and winter‐spawning groups (Figure 3). One of these peaks overlaid ESR2 (Figure 4c), an estrogen receptor. Tajima's *D* deviated from zero in both populations but was more negative in spring spawners than winter spawners (Figure 4c), although, again, the patterns were highly complex. There was also an increase in *d_xy_* in the region (Figure 4d). Further studies into these regions with higher sequencing coverage would be required to determine the ancestral states and could investigate whether the large, steep‐sided peak on LG18 is a chromosomal inversion.

**Figure 5:**
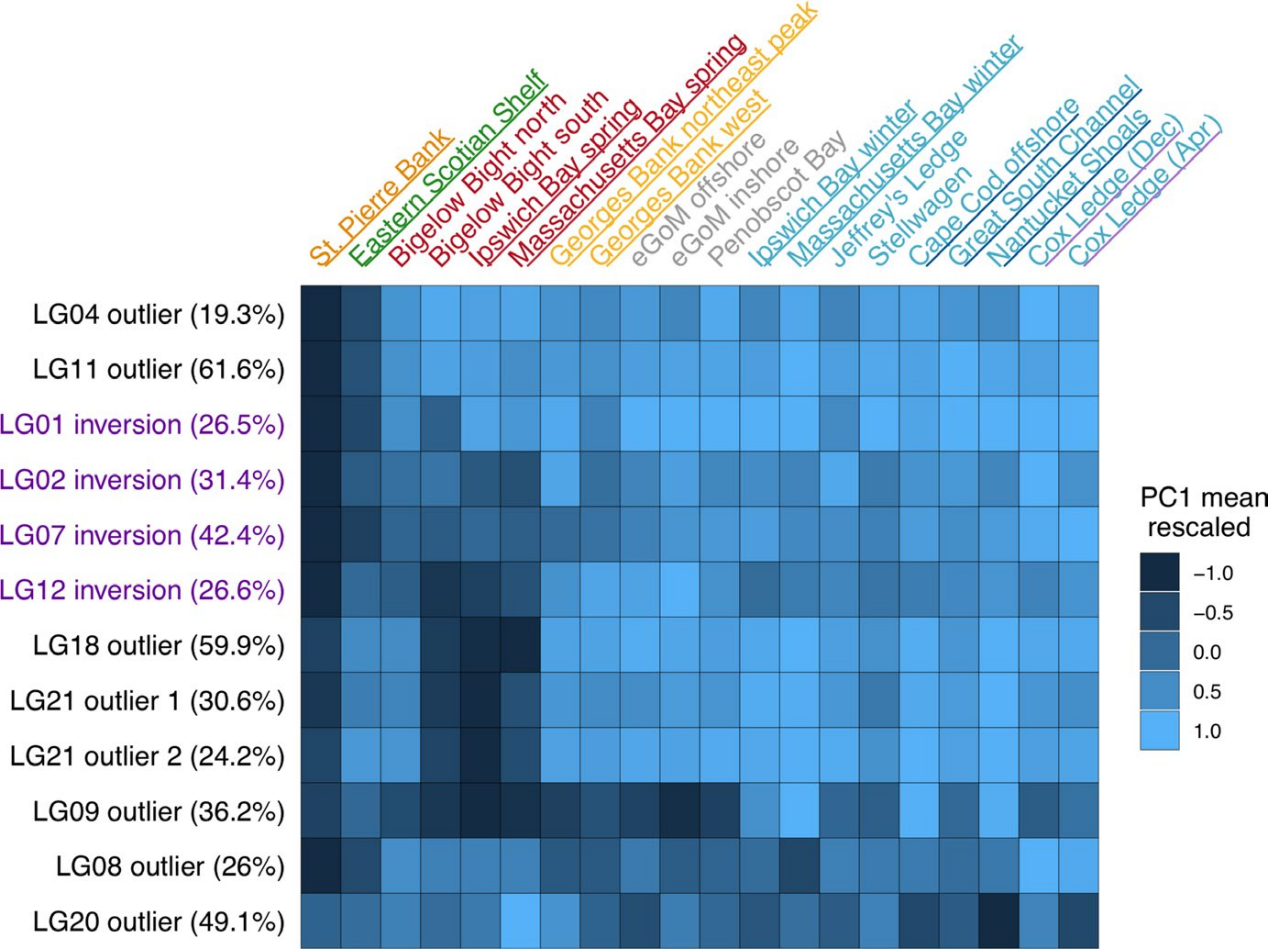
Heat map depicting how the chromosomal inversions and outlier regions of the genome differentiate among the 20 sampling locations included in the study. The labels of the sampling location are colored according to our expectations of the population structure: red and blue = northern spring coastal complex and southern complex, respectively, of Kovach et al. (2010); yellow = Georges Bank; green = eastern Scotian Shelf; orange = St. Pierre Bank; gray = eastern GoM. The color of the underlined labels indicates the sampling locations that were grouped for our pairwise Manhattan plots in Figure 3. Values in parentheses on the *y*‐axis are the amount of variation explained by PC1 in each individual‐level PCA

To synthesize how the inversions and outlier peaks differentiated among all the sampling locations included in the study, we created a heat map depicting the mean position of individuals from each sampling location along PC1 when SNPs within the outlier region were used for individual‐level PCA (Figure 5). The heat map illustrates how these regions of the genome differentiated sampling locations in different ways. For example, the outliers on LG 4 and 11 mainly separate the Canadian samples from U.S. samples. The inversions on LG 2, 7, and 12 group together a block of sampling locations at the left of the plot that includes the Canadian samples and spring‐spawning wGoM locations (Bigelow Bight north and south, Ipswich Bay spring, and Massachusetts Bay spring). Georges Bank and eGoM samples grouped together in another block on LG 12 where haplotype frequencies differed dramatically from those in the spring‐spawning sampling locations. This block was not seen on LG 2 and 7, however, where haplotype frequencies appeared to show more of a gradient between spring spawners on the left and the other U.S. sampling locations on the right. Another noticeable block was formed by the outliers on LG 18 and 21, which differentiated the spring‐spawning locations, including St. Pierre Bank and the spring spawners in the wGoM, except for Bigelow Bight north, from all other sampling locations. The outlier peak on LG 8, which overlies the heat shock proteins, mainly differentiated the southerly Cox Ledge sampling locations and, to some extent, the northern spring coastal complex from the Canadian samples, southern complex, and Georges Bank. This heat map revealed the complex patterns of putatively adaptive differentiation that exist among spawning locations.

**Figure 6:**
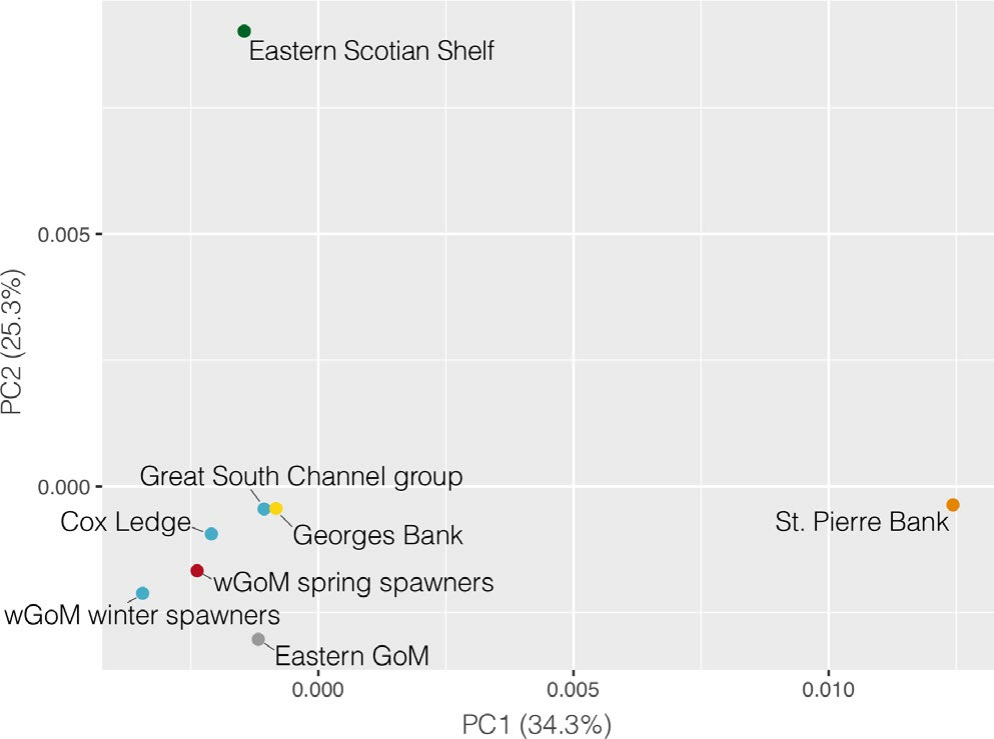
MDS plot showing the population structure based on the neutral SNP data set for PC1 and PC2. The colors represent our a priori understanding and expectations of the population structure: red and blue = northern spring coastal complex and southern complex, respectively, of Kovach et al. (2010); yellow = Georges Bank; green = eastern Scotian Shelf; orange = St. Pierre Bank; gray = eastern GoM

These patterns of putatively adaptive differentiation among groups did not match the patterns of putatively neutral differentiation. When only neutral SNPs were used to estimate pairwise *F_ST_* among groups, Canadian sampling locations appeared to be differentiated from one another and from the U.S. groups along PC1 and PC2 of the MDS (Figure 6), suggesting demographic separation of the two Canadian sampling locations. However, the U.S. sampling locations tended to cluster together on these PCs, suggesting only subtle neutral differentiation among U.S. groups. Georges Bank and the Great South Channel group appeared highly connected to one another, while Cox Ledge, wGoM spring spawners, wGoM winter spawners, and the eastern GoM appeared subtly differentiated from one another. This pattern was also evident on PC3 and PC4, which together captured the structure within the U.S groups (Figure S18). Along these axes, Cox Ledge, wGoM spring spawners, and wGoM winter spawners appeared subtly differentiated from one another and from Georges Bank, the Great South Channel Group, and the eastern GoM, which were similar to one another. Pairwise *F_ST_* values among groups based on the 5,579,519 putatively neutral loci are found in Table S12 while pairwise *F_ST_* among all sampling locations based on the 25,276 neutral, stringent loci can be found in Table S13. These *F_ST_* values among sampling locations are slightly higher than the values among groups as a result of fewer individuals in each sampling location, but the patterns remain the same. The individual‐level PCA among all sampling locations had little power to detect neutral genetic differentiation among sampling locations, highlighting the low levels of neutral differentiation that exist in this region (Figure S19).

## 4 DISCUSSION

Here, we present the first region‐wide analysis of population structure in Atlantic cod using whole‐genome sequencing. We found large haplotype frequency differences of the well‐known chromosomal inversions on LG 1, 2, 7, and 12 among sampling locations within U.S. and nearby Canadian waters. However, we also found multiple, previously undescribed outlier peaks outside of these inversions that also contributed significantly to population structure, sometimes showing very high levels of differentiation. Pairwise comparisons among sampling locations revealed complex patterns of population structure driven by outlier peaks and chromosomal inversions, suggesting that there are signals of multifaceted adaptation among sampled locations. In comparisons between allochronic spring‐ and winter‐spawning populations, we found multiple genes with reproductive functions associated with distinct outlier peaks, potentially shedding light on the mechanisms that determine timing of spawning in Atlantic cod.

It is not surprising that the haplotype frequency differences at the inversions were key drivers of population structure in this genome‐wide SNP data set since they encompass large tracts of the genome (around 7% in total). The magnitude of the haplotype frequency differences between our U.S. and Canadian samples were significant against low, neutral differentiation, suggesting that selective pressures acting on these inversions are likely important for local adaptation (Figure 3). The inversions were not found to be in inter‐chromosomal linkage disequilibrium, contrary to previous findings (Bradbury et al., 2014), and, at the population level, they displayed differing effects on population structure (Figures 2 and 3). The inversions on LG 1 differed in haplotype frequencies almost exclusively in comparisons between U.S. and Canadian samples. In addition to life‐history divergence between migratory and stationary ecotypes in the Northeast Atlantic and in Canada (Berg et al., 2017, 2016; Hemmer‐Hansen et al., 2013; Kirubakaran et al., 2016; Sinclair‐Waters et al., 2017), LG1 inversions are thought to be linked to temperature, as noted previously for the NW Atlantic (Bradbury et al., 2013; Sinclair‐Waters et al., 2017) and Greenland (Therkildsen, Hemmer‐Hansen, Hedeholm, et al., 2013). Supporting this, our two Canadian samples were from north of the biogeographic break found on the Scotian Shelf (Stanley et al., 2018) that separates the cooler Canadian shelf waters from the warmer U.S. waters. In the NW Atlantic, selection pressures on the inversions on LG 2, 7, and 12 have also been linked to temperature (Barney et al., 2017; Berg et al., 2017; Bradbury et al., 2010). We found similar haplotype frequencies for these inversions among the Canadian samples and the spring‐spawning samples in the western GoM, suggesting that spring‐spawning GoM cod may have adaptations and life‐history strategies associated with cooler temperatures than winter‐spawning populations. It is thought that spring‐spawned juveniles settle at greater depths (<80 m) and a narrower range of temperatures (<10°C) than winter‐spawned juveniles (<30 m, 5–15°C; Howe, Correia, Currier, King, & Johnston, 2002; M. Dean, Massachusetts Division of Marine Fisheries, pers. comm.). Depth and/or temperature could therefore maintain selection pressures on these inversions creating the haplotype frequency differences that we observed, but further research would be necessary to confirm this.

Outside of the inversions, pairwise comparisons between spring‐spawning cod (St. Pierre Bank and wGoM spring spawners) and winter‐spawning cod (all other groups) revealed strong peaks of genetic differentiation that likely underlie the mechanisms regulating timing of spawning (Figure 3, Table 1), potentially contributing to prezygotic isolation among these spawning groups. We observed large peaks in both relative (*F_ST_*) and absolute (*d_xy_*) genetic differentiation on LG 18 in a region surrounding the FSHR gene. This gene codes for a receptor for follicle‐stimulating hormone (FSH) and may also bind luteinizing hormone (LH) (Swanson, Dickey, & Campbell, 2003). FSH and LH, secreted by the pituitary gland, have well‐established roles in the regulation of gametogenesis in fish (Swanson et al., 2003) and can trigger reproduction in response to changes in photoperiod (Bromage, Whitehead, & Breton, 1982; Choi, Lee, Park, Kim, & Sohn, 2010; Davie, Porter, Bromage, & Migaud, 2007; Hansen et al., 2001; Migaud, Davie, & Taylor, 2010; Peter & Crim, 1979). In female fish, FSH promotes the secretion from ovarian follicles of estradiol‐17β, an estrogen steroid hormone. We found significant sequence divergence associated with ESR2, an estrogen receptor, on LG 21 and an *F_ST_* peak surrounding HSD17B2 on LG 9, which catalyses the conversion of oestradiol to oestrone and testosterone to androstenedione. In Atlantic herring (*Clupea harengus*), ESR2 has similarly been associated with timing of spawning in spring and autumn‐spawning populations (Lamichhaney et al., 2017). Taken together, this provides strong evidence that the brain‐pituitary‐gonadal axis is likely involved in regulating timing of spawning, possibly through photoperiod, and these genes may contribute to prezygotic isolation of winter‐ and spring‐spawning cod. We also found an *F_ST_* outlier peak associated with SYNE2, as did Lamichhaney et al., (2017) in Atlantic herring, on LG 21 close to ESR2. SYNE2 has no known role in reproduction, but it appears to be linked to spawning timing in both herring and cod, either through hitherto unknown pathways or perhaps because it is close to long‐range regulatory elements associated with ESR2 as postulated by Lamichhaney et al. (2017). Finally, we observed an *F_ST_* peak with a gene linked to reproduction on LG 20; NME8 is thought to be involved in sperm tail maturation and showed elevated differentiation between wGoM spring spawners and the Great South Channel group, which are winter spawners. Overall, these findings suggest that multiple genes could be involved in regulating reproduction in these allochronic spawning populations, some of which show parallel adaptations across geographically distant populations and species. Notably, many of our peaks related to spawning timing were relatively narrow (range 30–500 kb) and may have been difficult to detect with low‐density SNP screening, highlighting a key advantage of the low‐coverage, full genome sequencing approach used here (Therkildsen & Palumbi, 2017).

The absolute genetic differentiation (*d_xy_*) at the peaks on LG18 and LG21 suggests that these peaks have been targets of selection and may be resistant to introgression, possibly by contributing to prezygotic isolation between winter‐ and spring‐spawning cod. Prezygotic isolation does not evolve easily under gene flow as recombination tends to break up associations between traits involved in assortative mating and the preferences for those traits. However, prezygotic isolation can evolve under gene flow through a “one‐allele mechanism” whereby a single allele controls assortative mating (Felsenstein, 2006; Ortiz‐Barrientos & Noor, 2005; Servedio & Noor, 2003). The outlier peak on LG18 could act as one such one‐allele mechanism if it, alone, determines timing of spawning. The genetic differentiation we found at multiple other peaks linked to reproduction may suggest timing of spawning is instead controlled by multiple alleles or that these other peaks are subject to postzygotic selection. It is not clear whether differential timing of spawning evolved in allopatry (allowing for multiple allele control) or in sympatry (favouring a one‐allele mechanism) but follow‐up studies using higher coverage genomes should investigate the history of these populations. Secondary contact, producing similar patterns of heterogeneous differentiation throughout the genome, has been documented in other marine species (e.g., European sea bass, Duranton et al., 2018; European anchovies, Le Moan, Gagnaire, & Bonhomme, 2016) and could have occurred between spring‐ and winter‐spawning cod that evolved in allopatry.

Other peaks with gene functions unrelated to reproduction were also apparent in our pairwise comparisons (Figure 3, Table 1). For example, the comparison between St. Pierre Bank and Cox Ledge, at the latitudinal and thermal extremes of our sampling range, highlighted an *F_ST_* peak on LG 8 that included two heat shock protein (Hsp) coding genes (HSPB1 and HSPB8). These are both members of the small Hsp family and are known to interact with one another (Sun et al., 2003). Hsps are upregulated in fish in response to many types of stressors, including temperature and osmotic stress, as well as functioning in many aspects of physiology (Padmini, 2010). They have a critical role in helping fish cope with environmental change and are thought to be the primary mediators of thermal tolerance (Basu et al., 2002). HSPB1 has been linked to heat stress and thermal tolerance in zebrafish (*Danio rerio*; Mao, Bryantsev, Chechenova, & Shelden, 2005), rainbow trout (*Oncorhynchus mykiss*; Mosser & Bols, 1988), and various other marine taxa (e.g., Barshis et al., 2013; Tangwancharoen, Moy, & Burton, 2018). These Hsps are also known to be activated by estrogen and thus could have a reproductive role but, given that the outlier peak was most pronounced in comparisons between sampling locations from the most divergent thermal regimes in our sampled area, temperature may be driving selection. The lack of a peak in *d_xy_*, however, could instead suggest that this is a region of low recombination where background selection against deleterious mutations has created an *F_ST_* peak (Charlesworth, Morgan, & Charlesworth, 1993; Charlesworth, Nordborg, & Charlesworth, 1997). Alternatively, the peak in *F_ST_* may be the result of recent selection where too little time has passed for sequence divergence to accrue.

Variation in recombination rate throughout the genome can create *F_ST_* peaks (Burri et al., 2015; Cruickshank & Hahn, 2014; Ravinet et al., 2017) as shown, for example, in sticklebacks (Roesti, Hendry, Salzburger, & Berner, 2012) and European sea bass (Tine et al., 2014). Further investigation of the *F_ST_* peaks identified in this study is therefore warranted to determine the exact process underlying their creation. Furthermore, in the marine realm, local adaptation in quantitative traits is likely to evolve through polygenic architecture, creating high variation in allele effect size (Gagnaire & Gaggiotti, 2016). Our genome‐scan method will lack power to detect many relevant, small effect loci (Le Corre & Kremer, 2012; Pritchard & Di Rienzo, 2010) since subtle variations in allele frequencies will not be detected without very large sample sizes. Genome‐scan methods using windowed estimates of *F_ST_* may also miss targets of recent selection, since genetic differentiation around the target of selection will initially be limited to a narrow window (Ravinet et al., 2017). However, summary statistics estimated from low‐coverage sequencing datasets without very high sample sizes are noisy at the SNP‐level; targeted follow‐up work with larger sample sizes could yield further insights into the landscape of adaptive differentiation in Atlantic cod.

### 4.1 Implications for stock structure

**Figure 7:**
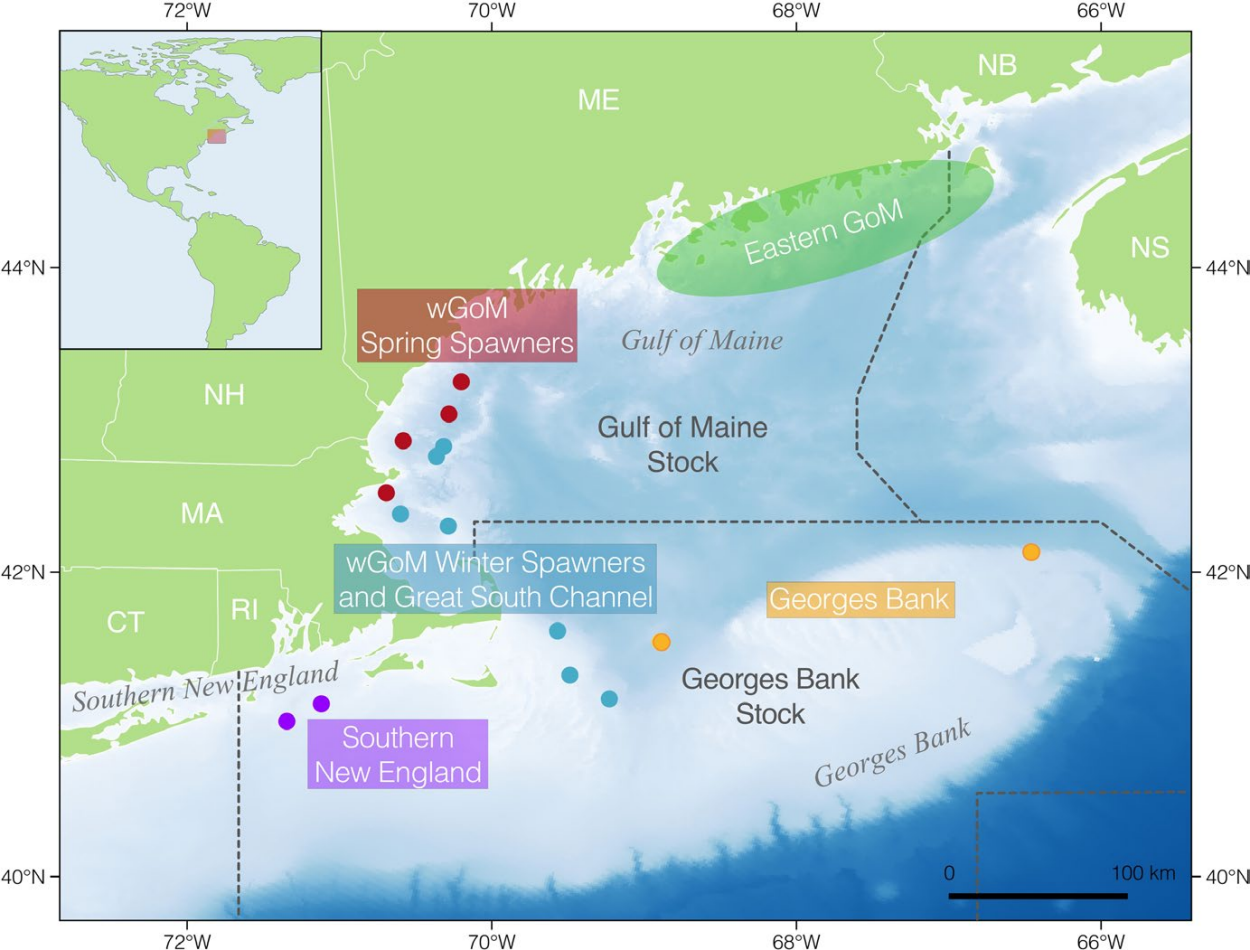
A map of the Gulf of Maine depicting our updated understanding of the population structure. Sampling locations are colored according to their membership of the main spawning complexes that we uncovered. Conclusions regarding the eastern GoM are preliminary as we did not have samples from spawning fish for this region. The gray dotted lines denote the boundaries of the Georges Bank stock and Gulf of Maine stock

The results presented here highlight the uniqueness of the wGoM spring spawners from the other U.S. spawning aggregations. We found large haplotype frequency differences at both chromosomal inversions and regions housing genes with reproductive functions (Figures 3, 4, 5) in addition to some subtle neutral differentiation of the spring spawners (Figure 6). It should be noted that neutral pairwise *F_ST_* values were all close to zero within U.S. waters (Tables S12 and S13), which is not unexpected based on previous studies (Barney et al., 2017; Clucas, Kerr, et al., 2019; Kovach et al., 2010), and is typical of findings from cod and other marine species. The interpretation of very small *F_ST_* is not straightforward (Conover et al., 2006; Li et al., 2018; Waples & Gaggiotti, 2006; Waples, Punt, & Cope, 2008), meaning it is not clear what the implications of the patterns of weak neutral differentiation may be for demographic connectivity of subpopulations. Nevertheless, the signals of putatively adaptive genetic differentiation between the wGoM spring spawners and other U.S. groups were significant. Yet, this differentiation is not recognized in current fisheries management structures (Figure 7).

Georges Bank appeared intermediate between the wGoM winter and spring spawners using the full SNP data set (Figure 2), consistent with previous findings (Clucas, Kerr, et al., 2019; Kovach et al., 2010) and showed similarity to the Great South Channel group at neutral markers (Figure 6, Figure S18) but not at putatively adaptive markers (Figure 3). Larval dispersal data suggest that a portion of larvae spawned on Georges Bank are likely swept westwards across the Great South Channel (Lough et al., 2006), likely explaining the low neutral differentiation, while postlarval selection may create the adaptive differentiation we found. We also showed that Georges Bank was distinct from Cox Ledge (Figure 2) with putative signals of selection at both chromosomal inversions and outlier peaks (Figures 3, 4, 5) in addition to some subtle neutral differentiation (Figure 6, Figure S18). Historical tagging studies (Wise, 1963) and larval circulation models (Churchill, Jeffrey, & Chen, 2011) have suggested little connectivity between cod in southern New England and Gulf of Maine waters. This, along with warmer ocean temperatures, may explain the genetic distinctiveness of Cox Ledge and its potential demographic independence from other U.S. sampling locations. These patterns are not reflected in current management structures; Cox Ledge and Great South Channel are managed as part of the Georges Bank stock (Figure 7). However, the genetic differentiation of both from Georges Bank should be considered in future management plans, especially under scenarios of rapid ocean warming since Cox Ledge showed genetic differentiation at a region of the genome housing heat shock proteins.

The eastern GoM nonspawning samples appeared most like the wGoM winter spawners with a very little putatively adaptive differentiation among them (Figure 2). However, they also shared some adaptive genetic variation with Georges Bank (Figure 5) and appeared to show the least amount of neutral differentiation from the Great South Channel group (Figure S18, Table S12). This finding is similar to previous work, that suggested connectivity between the eastern GoM and the western GoM, and yet similarity at adaptive loci between eastern GoM and Georges Bank cod (Clucas, Kerr, et al., 2019). Spawning aggregations have been absent from the eastern GoM in recent decades (Ames, 2004), and so it is unclear whether these nonspawning fish represent remnants from historical aggregations or recent migrants into eastern GoM. Tagging studies have shown movement between the western Scotian Shelf and both the eGoM and Georges Bank (Hunt, Stobo, & Almeida, 1999; Tallack, 2009), suggesting one possible route of connectivity. Additional samples from populations in the Bay of Fundy and Browns Bank would provide further insight into connectivity of the eGoM with surrounding populations. We summarize our understanding of the patterns of population structure, focusing on populations in U.S. waters, in Figure 7.

The complex patterns of population structure and putatively adaptive diversity among cod spawning populations that we show here are not recognized in current cod management units (Figure 7). Cod in U.S. waters is managed as two stocks: a Gulf of Maine stock and a Georges Bank stock that also includes cod in waters from southern New England to the mid‐Atlantic (Serchuk & Wigley, 1992). Under the current management structure, the Georges Bank stock represents a mixed stock comprising genetically differentiated groups of cod from Georges Bank, the Great South Channel area, and southern New England. The Gulf of Maine stock represents a mixed stock comprising the genetically distinct wGoM winter and spring spawners (Figure 7). Further, genetic similarity of cod spawning in the western GoM (Gulf of Maine stock) and the Great South Channel area (Georges Bank stock) indicates connectivity between the two stocks, as currently defined. A mismatch between this biological structure and the two‐stock management model may in part explain the failure of these stocks to recover despite decades of intensive management (Kerr, Cadrin, & Kovach, 2014).

The preservation of adaptive diversity is important for the conservation of threatened species (Funk et al., 2012) and necessary to support the resilience and recovery of fisheries to both exploitation and environmental perturbations (Hilborn et al., 2003; Kerr, Cadrin, & Secor, 2010a; Kerr et al., 2010b; Schindler et al., 2010). The wGoM spring spawners hold unique adaptive diversity within U.S. waters and may have some demographic separation. They are relatively limited in their range and could be at risk of extirpation if management structures are not updated to reflect their distinctiveness from the winter‐spawning population with which they overlap spatially. Furthermore, our results suggest winter and spring spawners may have different thermal tolerances, meaning they may show divergent responses to the observed and predicted extreme warming of the GoM (Pershing et al., 2015; Saba et al., 2016; Thomas et al., 2017). Managing these genetically distinct populations may be important for preserving evolutionary potential. Monitoring of fisheries induced mortality separately on these two populations is needed for sustainable management, and a set of SNPs for assigning catches to their population of origin could be identified for this purpose. In addition to the wGoM winter and spring spawners, Georges Bank, and Cox Ledge also represent spawning populations with distinct patterns of putatively adaptive genetic diversity. The adaptive differences among these populations suggests that if they became severely depleted, their potential to recover through migration from other populations would be limited, because a portion of immigrants may have low fitness (Peterson, Hilborn, & Hauser, 2014). Our findings provide a high‐resolution picture of population structure that should be used in revising the management of U.S. Atlantic cod stocks to preserve the putatively adaptive diversity of the remaining subpopulations. More broadly, our work also highlights the application of genome‐wide patterns of divergence and signatures of local adaptation in characterizing populations for informing management decisions.

## CONFLICT OF INTEREST

None declared.

## Data Availability

The genomic sequence data are archived in the NCBI Short Read Archive under BioProject ID PRJNA560242 (Clucas, Lou, Therkildsen, & Kovach, 2019).
