# Supplementary material for: Novel signals of adaptive genetic variation in northwestern Atlantic cod revealed by whole‐genome sequencing
Source: Evol Appl. 2019 Sep 13;12(10):1971–87. doi: 10.1111/eva.12861 (PMC6824067; doi:10.1111/eva.12861)
Supplement: Supplementary file 1 [file EVA-12-1971-s001.docx]

**­­**

Supporting Information

­­

**Novel signals of adaptive genetic variation in northwestern Atlantic cod revealed by whole genome sequencing**

Gemma V. Clucas, R. Nicolas Lou, Nina O. Therkildsen, and Adrienne I. Kovach.

Supplementary Table 1. Details of the sampling locations and the final number of individuals from each (N). The average sequencing depth across individuals in each sampling location is also given.

| Sampling location | Latitude | Longitude | N | Collection dates | Spawning season | Reproductive status | Average sequencing depth |
| --- | --- | --- | --- | --- | --- | --- | --- |
| St. Pierre Bank (3PS) | 46.00 | -55.60 | 11 | Spring 2010 | Spring | Unknown | 0.80 |
| Eastern Scotian Shelf (4VsW)* | 44.49 | -58.43 | 15 | Spring 2010 | Spring, summer, and fall | Unknown | 0.74 |
| Bigelow Bight north | 43.250 | -70.200 | 15 | Jul 2007 | Spring | Spent | 0.59 |
| Bigelow Bight south | 43.038 | -70.281 | 15 | Jul 2010 | Spring | Mostly spent | 0.98 |
| Ipswich Bay spring | 42.862 | -70.581 | 15 | May 2015 | Spring | Ripe & running | 0.53 |
| Massachusetts Bay spring | 42.520 | -70.692 | 14 | May 2013 | Spring | Ripe & running | 0.41 |
| Georges Bank northeast peak | Approx. 42.13 | Approx.  -66.46 | 25 | Feb 2006 & Feb 2007 | Winter | Ripe/ ripe & running/ spent | 0.68 |
| Georges Bank west | 41.542 | -68.894 | 15 | Jan – Mar 2010 | Winter | Spawning | 0.75 |
| eGoM offshore | Variable, see Fig. 1 | Variable, see Fig. 1 | 14 | Jul – Sep 2013 – 2015 | Unknown | Unknown | 0.57 |
| eGoM inshore | Variable, see Fig. 1 | Variable, see Fig. 1 | 15 | Oct 2008 & Jun – Oct 2013 – 2015 | Unknown | Unknown | 0.60 |
| Penobscot Bay | Variable, see Fig. 1 | Variable, see Fig. 1 | 14 | Oct 2008 & Jul – Oct 2013 – 2015 | Unknown | Unknown | 0.55 |
| Ipswich Bay winter | 42.826 | -70.316 | 14 | Dec 2014 | Winter | Ripe & running | 0.46 |
| Massachusetts Bay winter | 42.382 | -70.599 | 15 | Dec 2013 | Winter | Ripe/ ripe & running | 0.62 |
| Jeffrey's Ledge | 42.760 | -70.363 | 14 | Dec 2007 | Winter | Ripening/ ripe/ spawning | 0.67 |
| Stellwagen Bank | 42.303 | -70.285 | 23 | Mar 2006 & Mar 2007 | Early spring | Ripe/ ripe & running/ spent | 0.79 |
| Cape Cod offshore | 41.614 | -69.568 | 15 | Dec 2009 | Fall | Ripe/ spawning | 0.92 |
| Great South Channel | 41.168 | -69.228 | 12 | Jan – Mar 2010 | Fall | Ripening/ spawning | 0.62 |
| Nantucket Shoals | 41.324 | -69.487 | 15 | Nov 2006 | Fall | Ripe/ spawning | 0.67 |
| Cox Ledge (Dec) | 41.135 | -71.119 | 15 | Jan 2007 | Winter | Ripe/ spawning | 0.70 |
| Cox Ledge (Apr) | 41.020 | -71.345 | 15 | Apr 2007 | Early spring | Ripe/ spawning | 0.71 |

*** Latitude and longitude are the mean of multiple collections on the eastern Scotian Shelf.

Supplementary Table 2. Weighted pairwise F_ST_ values between all sampling locations, calculated with the stringent SNP data set.

|  | **St. Pierre Bank** | **Eastern Scotian Shelf** | **Bigelow Bight north** | **Cape Cod offshore** | **Cox Ledge (Apr)** | **Cox Ledge (Dec)** | **Georges Bank northeast peak** | **Georges Bank west** | **Great South Channel** | **Ipswich Bay spring** | **Ipswich Bay winter** | **Jeffrey’s Ledge** | **Massachusetts Bay spring** | **Massachusetts Bay winter** | **Nantucket Shoals** | **eGom inshore** | **eGoM offshore** | **Penobscot Bay** | **Stellwagen Bank** | **Bigelow Bight south** |
| --- | --- | --- | --- | --- | --- | --- | --- | --- | --- | --- | --- | --- | --- | --- | --- | --- | --- | --- | --- | --- |
| **St. Pierre Bank** | *** | 0.016 | 0.031 | 0.05 | 0.062 | 0.059 | 0.034 | 0.036 | 0.049 | 0.033 | 0.053 | 0.045 | 0.030 | 0.049 | 0.051 | 0.055 | 0.045 | 0.051 | 0.042 | 0.026 |
| **Eastern Scotian Shelf** | 0.016 | *** | 0.018 | 0.032 | 0.039 | 0.038 | 0.019 | 0.02 | 0.031 | 0.021 | 0.035 | 0.027 | 0.019 | 0.031 | 0.031 | 0.032 | 0.027 | 0.031 | 0.026 | 0.018 |
| **Bigelow Bight north** | 0.031 | 0.018 | *** | 0.018 | 0.022 | 0.02 | 0.011 | 0.013 | 0.016 | 0.013 | 0.019 | 0.016 | 0.014 | 0.019 | 0.016 | 0.019 | 0.015 | 0.017 | 0.013 | 0.014 |
| **Cape Cod offshore** | 0.05 | 0.032 | 0.018 | *** | 0.013 | 0.014 | 0.013 | 0.016 | 0.013 | 0.018 | 0.015 | 0.014 | 0.023 | 0.015 | 0.012 | 0.014 | 0.014 | 0.013 | 0.012 | 0.022 |
| **Cox Ledge (Apr)** | 0.062 | 0.039 | 0.022 | 0.013 | *** | 0.013 | 0.017 | 0.019 | 0.014 | 0.023 | 0.016 | 0.015 | 0.029 | 0.018 | 0.013 | 0.014 | 0.016 | 0.014 | 0.015 | 0.03 |
| **Cox Ledge (Dec)** | 0.059 | 0.038 | 0.02 | 0.014 | 0.013 | *** | 0.016 | 0.018 | 0.015 | 0.022 | 0.015 | 0.015 | 0.027 | 0.017 | 0.014 | 0.014 | 0.015 | 0.014 | 0.014 | 0.028 |
| **Georges Bank northeast peak** | 0.034 | 0.019 | 0.011 | 0.013 | 0.017 | 0.016 | *** | 0.009 | 0.013 | 0.013 | 0.017 | 0.013 | 0.014 | 0.015 | 0.013 | 0.013 | 0.011 | 0.013 | 0.01 | 0.015 |
| **Georges Bank west** | 0.036 | 0.02 | 0.013 | 0.016 | 0.019 | 0.018 | 0.009 | *** | 0.015 | 0.017 | 0.019 | 0.014 | 0.017 | 0.017 | 0.015 | 0.014 | 0.013 | 0.015 | 0.012 | 0.019 |
| **Great South Channel** | 0.049 | 0.031 | 0.016 | 0.013 | 0.014 | 0.015 | 0.013 | 0.015 | *** | 0.018 | 0.015 | 0.014 | 0.021 | 0.015 | 0.012 | 0.014 | 0.014 | 0.014 | 0.012 | 0.022 |
| **Ipswich Bay spring** | 0.033 | 0.021 | 0.013 | 0.018 | 0.023 | 0.022 | 0.013 | 0.017 | 0.018 | *** | 0.02 | 0.018 | 0.013 | 0.018 | 0.019 | 0.021 | 0.017 | 0.018 | 0.014 | 0.013 |
| **Ipswich Bay winter** | 0.053 | 0.035 | 0.019 | 0.015 | 0.016 | 0.015 | 0.017 | 0.019 | 0.015 | 0.02 | *** | 0.016 | 0.023 | 0.017 | 0.014 | 0.016 | 0.017 | 0.015 | 0.014 | 0.026 |
| **Jeffrey’s Ledge** | 0.045 | 0.027 | 0.016 | 0.014 | 0.015 | 0.015 | 0.013 | 0.014 | 0.014 | 0.018 | 0.016 | *** | 0.021 | 0.016 | 0.013 | 0.014 | 0.014 | 0.014 | 0.013 | 0.021 |
| **Massachusetts Bay spring** | 0.03 | 0.019 | 0.014 | 0.023 | 0.029 | 0.027 | 0.014 | 0.017 | 0.021 | 0.013 | 0.023 | 0.021 | *** | 0.023 | 0.023 | 0.024 | 0.02 | 0.021 | 0.017 | 0.015 |
| **Massachusetts Bay winter** | 0.049 | 0.031 | 0.019 | 0.015 | 0.018 | 0.017 | 0.015 | 0.017 | 0.015 | 0.018 | 0.017 | 0.016 | 0.023 | *** | 0.015 | 0.017 | 0.017 | 0.016 | 0.014 | 0.024 |
| **Nantucket Shoals** | 0.051 | 0.031 | 0.016 | 0.012 | 0.013 | 0.014 | 0.013 | 0.015 | 0.012 | 0.019 | 0.014 | 0.013 | 0.023 | 0.015 | *** | 0.013 | 0.013 | 0.013 | 0.012 | 0.024 |
| **eGom inshore** | 0.055 | 0.032 | 0.019 | 0.014 | 0.014 | 0.014 | 0.013 | 0.014 | 0.014 | 0.021 | 0.016 | 0.014 | 0.024 | 0.017 | 0.013 | *** | 0.009 | 0.013 | 0.013 | 0.027 |
| **eGoM offshore** | 0.045 | 0.027 | 0.015 | 0.014 | 0.016 | 0.015 | 0.011 | 0.013 | 0.014 | 0.017 | 0.017 | 0.014 | 0.02 | 0.017 | 0.013 | 0.009 | *** | 0.014 | 0.012 | 0.022 |
| **Penobscot Bay** | 0.051 | 0.031 | 0.017 | 0.013 | 0.014 | 0.014 | 0.013 | 0.015 | 0.014 | 0.018 | 0.015 | 0.014 | 0.021 | 0.016 | 0.013 | 0.013 | 0.014 | *** | 0.013 | 0.024 |
| **Stellwagen Bank** | 0.042 | 0.026 | 0.013 | 0.012 | 0.015 | 0.014 | 0.01 | 0.012 | 0.012 | 0.014 | 0.014 | 0.013 | 0.017 | 0.014 | 0.012 | 0.013 | 0.012 | 0.013 | *** | 0.018 |
| **Bigelow Bight south** | 0.026 | 0.018 | 0.014 | 0.022 | 0.030 | 0.028 | 0.015 | 0.019 | 0.022 | 0.013 | 0.026 | 0.021 | 0.015 | 0.024 | 0.024 | 0.027 | 0.022 | 0.024 | 0.018 | *** |

Supplementary Table 3. Estimates of inter-chromosomal linkage disequilibrium in the St. Pierre Bank samples. The correlation coefficient is above the diagonal and the associated p-value is below the diagonal. The inversions on LG 2 and 7 were fixed for this sampling location.

|  | LG01 | LG12 |
| --- | --- | --- |
| LG01 | *** | 0.069 |
| LG12 | 0.219 | *** |

Supplementary Table 4. Estimates of inter-chromosomal linkage disequilibrium in the eastern Scotian Shelf samples. Correlation coefficients are above the diagonal and the associated p-values are below the diagonal. Values significant at the 𝛼 = 0.05 level are shown in bold.

|  | LG01 | LG02 | LG07 | LG12 |
| --- | --- | --- | --- | --- |
| LG01 | *** | **0.214** | 0.100 | 0.107 |
| LG02 | **0.011** | *** | 0.086 | 0.014 |
| LG07 | 0.084 | 0.109 | *** | 0.072 |
| LG12 | 0.074 | 0.523 | 0.143 | *** |

Supplementary Table 5. Estimates of inter-chromosomal linkage disequilibrium in the western GoM spring spawning samples. Correlation coefficients are above the diagonal and the associated p-values are below the diagonal. Values significant at the 𝛼 = 0.05 level are shown in bold.

|  | LG01 | LG02 | LG07 | LG12 |
| --- | --- | --- | --- | --- |
| LG01 | *** | 0.002 | 0.000 | **0.044** |
| LG02 | 0.659 | *** | 0.000 | **0.040** |
| LG07 | 0.934 | 0.929 | *** | 0.000 |
| LG12 | **0.023** | **0.030** | 0.933 | *** |

Supplementary Table 6. Estimates of inter-chromosomal linkage disequilibrium in the western GoM winter spawning samples. Correlation coefficients are above the diagonal and the associated p-values are below the diagonal. Values significant at the 𝛼 = 0.05 level are shown in bold. The inversions on LG 1 were fixed for this sampling group.

|  | LG02 | LG07 | LG12 |
| --- | --- | --- | --- |
| LG02 | *** | 0.006 | **0.069** |
| LG07 | 0.560 | *** | **0.137** |
| LG12 | **0.046** | **0.005** | *** |

Supplementary Table 7. Estimates of inter-chromosomal linkage disequilibrium in the Great South Channel group. Correlation coefficients are above the diagonal and the associated p-values are below the diagonal.

|  | LG01 | LG02 | LG07 | LG12 |
| --- | --- | --- | --- | --- |
| LG01 | *** | 0.007 | 0.003 | 0.024 |
| LG02 | 0.436 | *** | 0.001 | 0.023 |
| LG07 | 0.594 | 0.827 | *** | 0.020 |
| LG12 | 0.158 | 0.166 | 0.199 | *** |

Supplementary Table 8. Estimates of inter-chromosomal linkage disequilibrium in the Georges Bank samples. Correlation coefficients are above the diagonal and the associated p-values are below the diagonal.

|  | LG01 | LG02 | LG07 | LG12 |
| --- | --- | --- | --- | --- |
| LG01 | *** | 0.025 | 0.008 | 0.015 |
| LG02 | 0.154 | *** | 0.007 | 0.036 |
| LG07 | 0.423 | 0.458 | *** | 0.031 |
| LG12 | 0.278 | 0.088 | 0.114 | *** |

Supplementary Table 9. Estimates of inter-chromosomal linkage disequilibrium in the Cox Ledge group. Correlation coefficients are above the diagonal and the associated p-values are below the diagonal. Values significant at the 𝛼 = 0.05 level are shown in bold. The inversions on LG 1 were fixed for this sampling group.

|  | LG02 | LG07 | LG12 |
| --- | --- | --- | --- |
| LG02 | *** | 0.036 | **0.071** |
| LG07 | 0.143 | *** | 0.003 |
| LG12 | **0.039** | 0.664 | *** |

Supplementary Table 10. Estimates of inter-chromosomal linkage disequilibrium in the eastern GoM group. Correlation coefficients are above the diagonal and the associated p-values are below the diagonal. Values significant at the 𝛼 = 0.05 level are shown in bold. The inversions on LG 1 were fixed for this sampling group.

|  | LG02 | LG07 | LG12 |
| --- | --- | --- | --- |
| LG02 | *** | **0.069** | 0.009 |
| LG07 | **0.015** | *** | 0.024 |
| LG12 | 0.380 | 0.153 | *** |

Supplementary Table 11. Estimates of the boundaries of chromosomal inversions calculated using ngsLD. Estimates may differ from those made in previous papers as a result of chromosomal rearrangements unique to this geographic sampling area or because of low densities of SNPs near the inversion boundaries. The inversion on LG01 comprises two adjacent inversions, the outer boundaries of which we report.

| Chromosome | Inversion start (Mb) | Inversion end (Mb) |
| --- | --- | --- |
| LG01 | 9.114 | 26.197 |
| LG02 | 18.494 | 24.053 (end of chromosome) |
| LG07 | 13.605 | 23.003 |
| LG12 | Approx. 0.5Mb but the boundary was not well-defined. | 13.406 |

Supplementary Table 12. Weighted pairwise F_ST_ values between groups, calculated with the neutral SNP data set.

|  | St. Pierre Bank | Eastern Scotian Shelf | wGoM spring spawners | wGoM winter spawners | Great South Channel group | Cox Ledge | Georges Bank | Eastern GoM |
| --- | --- | --- | --- | --- | --- | --- | --- | --- |
| St. Pierre Bank | *** | 0.0168 | 0.0158 | 0.0166 | 0.0140 | 0.0153 | 0.0138 | 0.0145 |
| Eastern Scotian Shelf | 0.0168 | *** | 0.0119 | 0.0124 | 0.0102 | 0.0111 | 0.0101 | 0.0125 |
| wGoM spring spawners | 0.0158 | 0.0119 | *** | 0.0089 | 0.0070 | 0.0080 | 0.0067 | 0.0071 |
| wGoM winter spawners | 0.0166 | 0.0124 | 0.0089 | *** | 0.0070 | 0.0079 | 0.0075 | 0.0079 |
| Great South Channel group | 0.0140 | 0.0102 | 0.0070 | 0.0070 | *** | 0.0059 | 0.0054 | 0.0056 |
| Cox Ledge | 0.0153 | 0.0111 | 0.0080 | 0.0079 | 0.0059 | *** | 0.0061 | 0.0066 |
| Georges Bank | 0.0138 | 0.0101 | 0.0067 | 0.0075 | 0.0054 | 0.0061 | *** | 0.0054 |
| Eastern GoM | 0.0145 | 0.0125 | 0.0071 | 0.0079 | 0.0056 | 0.0066 | 0.0054 | *** |

*Supplementary Table 12. Weighted pairwise F_ST_ values between all sampling locations, calculated with the neutral, stringent SNP dataset.*

|  | **St. Pierre Bank** | **Eastern Scotian Shelf** | **Bigelow Bight north** | **Cape Cod offshore** | **Cox Ledge (Apr)** | **Cox Ledge (Dec)** | **Georges Bank northeast peak** | **Georges Bank west** | **Great South Channel** | **Ipswich Bay spring** | **Ipswich Bay winter** | **Jeffrey’s Ledge** | **Massachusetts Bay spring** | **Massachusetts Bay winter** | **Nantucket Shoals** | **eGom inshore** | **eGoM offshore** | **Penobscot Bay** | **Stellwagen Bank** | **Bigelow Bight south** |
| --- | --- | --- | --- | --- | --- | --- | --- | --- | --- | --- | --- | --- | --- | --- | --- | --- | --- | --- | --- | --- |
| **St. Pierre Bank** | *** | 0.011 | 0.013 | 0.013 | 0.015 | 0.012 | 0.011 | 0.014 | 0.016 | 0.013 | 0.017 | 0.015 | 0.016 | 0.017 | 0.014 | 0.016 | 0.013 | 0.015 | 0.013 | 0.015 |
| **Eastern Scotian Shelf** | 0.011 | *** | 0.011 | 0.011 | 0.011 | 0.011 | 0.008 | 0.011 | 0.014 | 0.012 | 0.015 | 0.012 | 0.012 | 0.014 | 0.011 | 0.012 | 0.010 | 0.013 | 0.011 | 0.014 |
| **Bigelow Bight north** | 0.013 | 0.011 | *** | 0.011 | 0.011 | 0.011 | 0.009 | 0.010 | 0.013 | 0.011 | 0.015 | 0.011 | 0.013 | 0.016 | 0.010 | 0.012 | 0.011 | 0.013 | 0.011 | 0.012 |
| **Cape Cod offshore** | 0.013 | 0.011 | 0.011 | *** | 0.011 | 0.011 | 0.008 | 0.012 | 0.012 | 0.011 | 0.014 | 0.012 | 0.014 | 0.013 | 0.012 | 0.012 | 0.012 | 0.012 | 0.011 | 0.012 |
| **Cox Ledge (Apr)** | 0.015 | 0.011 | 0.011 | 0.011 | *** | 0.011 | 0.009 | 0.011 | 0.012 | 0.011 | 0.014 | 0.011 | 0.014 | 0.014 | 0.010 | 0.012 | 0.011 | 0.012 | 0.011 | 0.013 |
| **Cox Ledge (Dec)** | 0.012 | 0.011 | 0.011 | 0.011 | 0.011 | *** | 0.008 | 0.010 | 0.012 | 0.011 | 0.013 | 0.012 | 0.012 | 0.013 | 0.011 | 0.011 | 0.010 | 0.012 | 0.010 | 0.012 |
| **Georges Bank northeast peak** | 0.011 | 0.008 | 0.009 | 0.008 | 0.009 | 0.008 | *** | 0.009 | 0.010 | 0.008 | 0.012 | 0.010 | 0.010 | 0.012 | 0.009 | 0.009 | 0.009 | 0.010 | 0.008 | 0.009 |
| **Georges Bank west** | 0.014 | 0.011 | 0.010 | 0.012 | 0.011 | 0.010 | 0.009 | *** | 0.012 | 0.011 | 0.014 | 0.012 | 0.012 | 0.014 | 0.010 | 0.011 | 0.012 | 0.012 | 0.010 | 0.012 |
| **Great South Channel** | 0.016 | 0.014 | 0.013 | 0.012 | 0.012 | 0.012 | 0.010 | 0.012 | *** | 0.013 | 0.014 | 0.013 | 0.014 | 0.014 | 0.012 | 0.013 | 0.012 | 0.014 | 0.012 | 0.013 |
| **Ipswich Bay spring** | 0.013 | 0.012 | 0.011 | 0.011 | 0.011 | 0.011 | 0.008 | 0.011 | 0.013 | *** | 0.015 | 0.012 | 0.013 | 0.013 | 0.011 | 0.012 | 0.010 | 0.012 | 0.010 | 0.011 |
| **Ipswich Bay winter** | 0.017 | 0.015 | 0.015 | 0.014 | 0.014 | 0.013 | 0.012 | 0.014 | 0.014 | 0.015 | *** | 0.015 | 0.015 | 0.018 | 0.012 | 0.013 | 0.014 | 0.015 | 0.013 | 0.015 |
| **Jeffrey’s Ledge** | 0.015 | 0.012 | 0.011 | 0.012 | 0.011 | 0.012 | 0.010 | 0.012 | 0.013 | 0.012 | 0.015 | *** | 0.014 | 0.014 | 0.011 | 0.013 | 0.013 | 0.013 | 0.011 | 0.012 |
| **Massachusetts Bay spring** | 0.016 | 0.012 | 0.013 | 0.014 | 0.014 | 0.012 | 0.010 | 0.012 | 0.014 | 0.013 | 0.015 | 0.014 | *** | 0.017 | 0.015 | 0.013 | 0.014 | 0.014 | 0.012 | 0.013 |
| **Massachusetts Bay winter** | 0.017 | 0.014 | 0.016 | 0.013 | 0.014 | 0.013 | 0.012 | 0.014 | 0.014 | 0.013 | 0.018 | 0.014 | 0.017 | *** | 0.013 | 0.014 | 0.015 | 0.015 | 0.014 | 0.015 |
| **Nantucket Shoals** | 0.014 | 0.011 | 0.010 | 0.012 | 0.010 | 0.011 | 0.009 | 0.010 | 0.012 | 0.011 | 0.012 | 0.011 | 0.015 | 0.013 | *** | 0.012 | 0.011 | 0.011 | 0.010 | 0.013 |
| **eGom inshore** | 0.016 | 0.012 | 0.012 | 0.012 | 0.012 | 0.011 | 0.009 | 0.011 | 0.013 | 0.012 | 0.013 | 0.013 | 0.013 | 0.014 | 0.012 | *** | 0.009 | 0.013 | 0.011 | 0.013 |
| **eGoM offshore** | 0.013 | 0.010 | 0.011 | 0.012 | 0.011 | 0.010 | 0.009 | 0.012 | 0.012 | 0.010 | 0.014 | 0.013 | 0.014 | 0.015 | 0.011 | 0.009 | *** | 0.014 | 0.011 | 0.012 |
| **Penobscot Bay** | 0.015 | 0.013 | 0.013 | 0.012 | 0.012 | 0.012 | 0.010 | 0.012 | 0.014 | 0.012 | 0.015 | 0.013 | 0.014 | 0.015 | 0.011 | 0.013 | 0.014 | *** | 0.012 | 0.015 |
| **Stellwagen Bank** | 0.013 | 0.011 | 0.011 | 0.011 | 0.011 | 0.010 | 0.008 | 0.010 | 0.012 | 0.010 | 0.013 | 0.011 | 0.012 | 0.014 | 0.010 | 0.011 | 0.011 | 0.012 | *** | 0.011 |
| **Bigelow Bight south** | 0.015 | 0.014 | 0.012 | 0.012 | 0.013 | 0.012 | 0.009 | 0.012 | 0.013 | 0.011 | 0.015 | 0.012 | 0.013 | 0.015 | 0.013 | 0.013 | 0.012 | 0.015 | 0.011 | *** |

Supplementary Figure 1. The distribution of SNP sequencing depths for the full SNP data set when summed across all individuals. The orange dashed line indicates the mean depth.


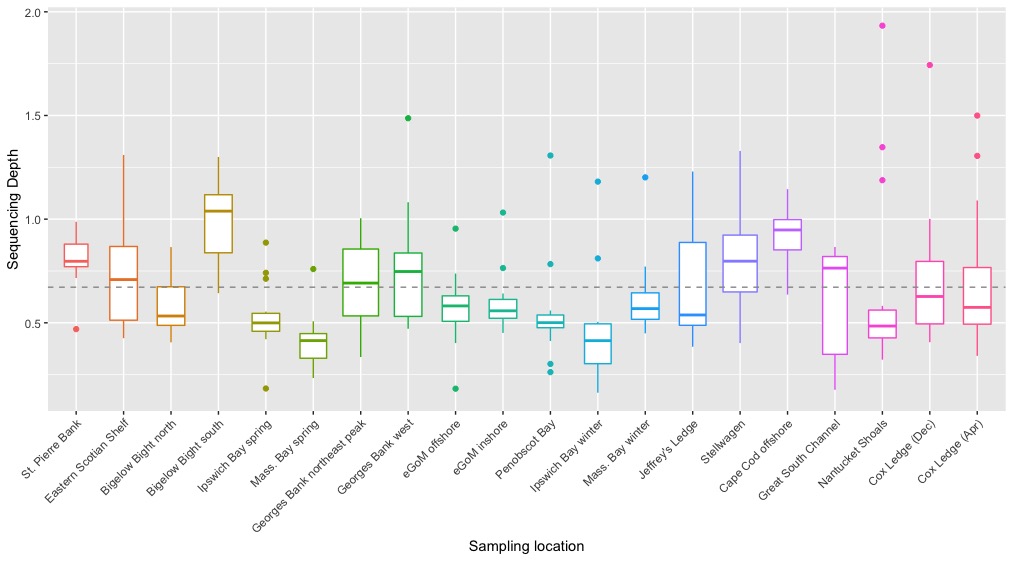


Supplementary Figure 2. Box plots showing the distribution of individual sequencing depth for each sampling location included in the study across all sites in the genome (i.e. including sites with zero depth). The horizontal dashed line represents the mean depth across all individuals while the width of each box represents the relative number of individuals in that sampling location.


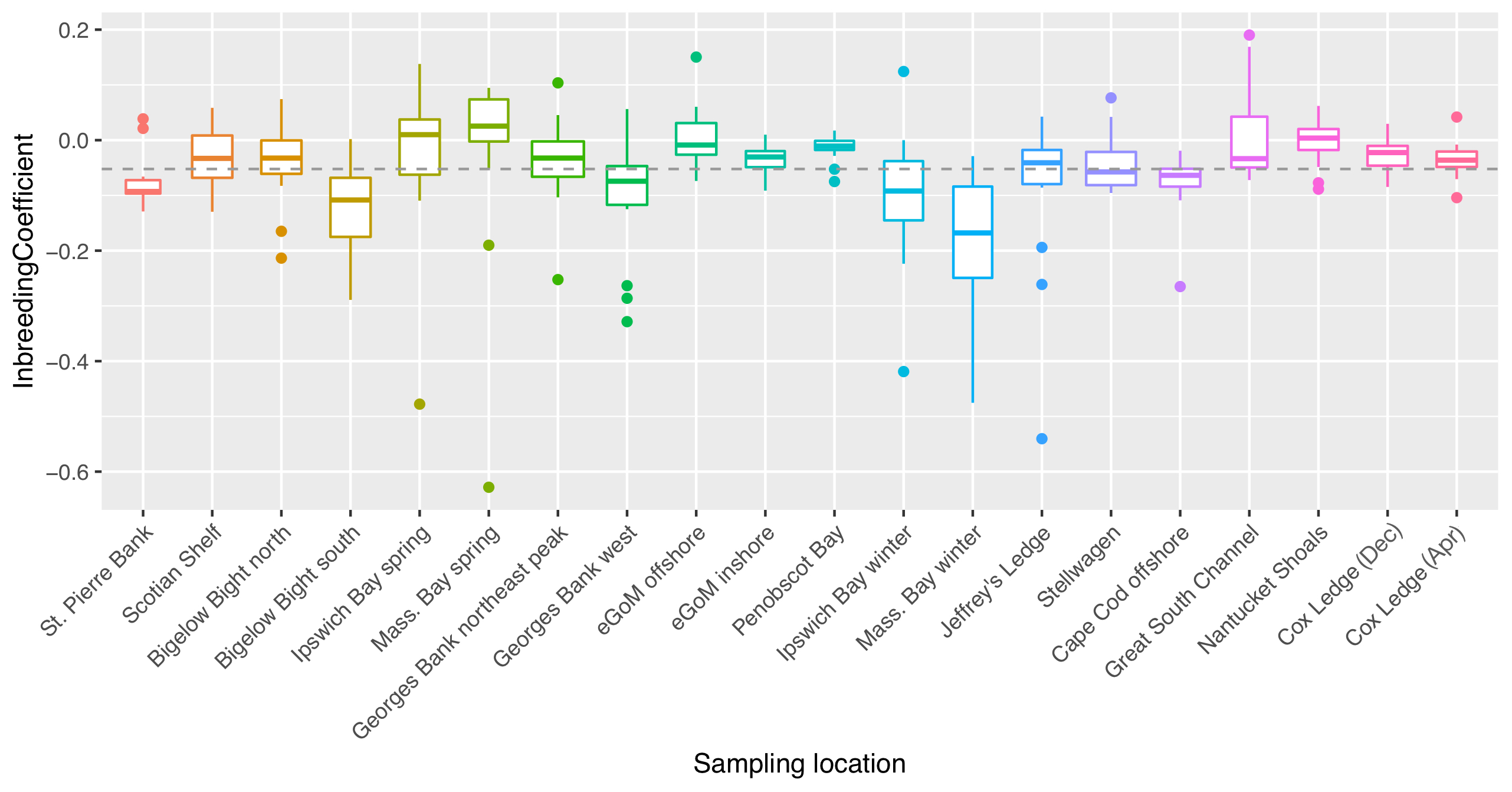


Supplementary Figure 3. Boxplots showing the distribution of individual inbreeding coefficients within each sampling location. The horizontal dashed line represents the mean inbreeding coefficients across all individuals while the width of each box represents the relative number of individuals in that sampling location

.

Supplementary Figure 4. The distribution of SNP sequencing depths for the stringent SNP data set when summed across all individuals. The orange dashed line indicates the mean depth.


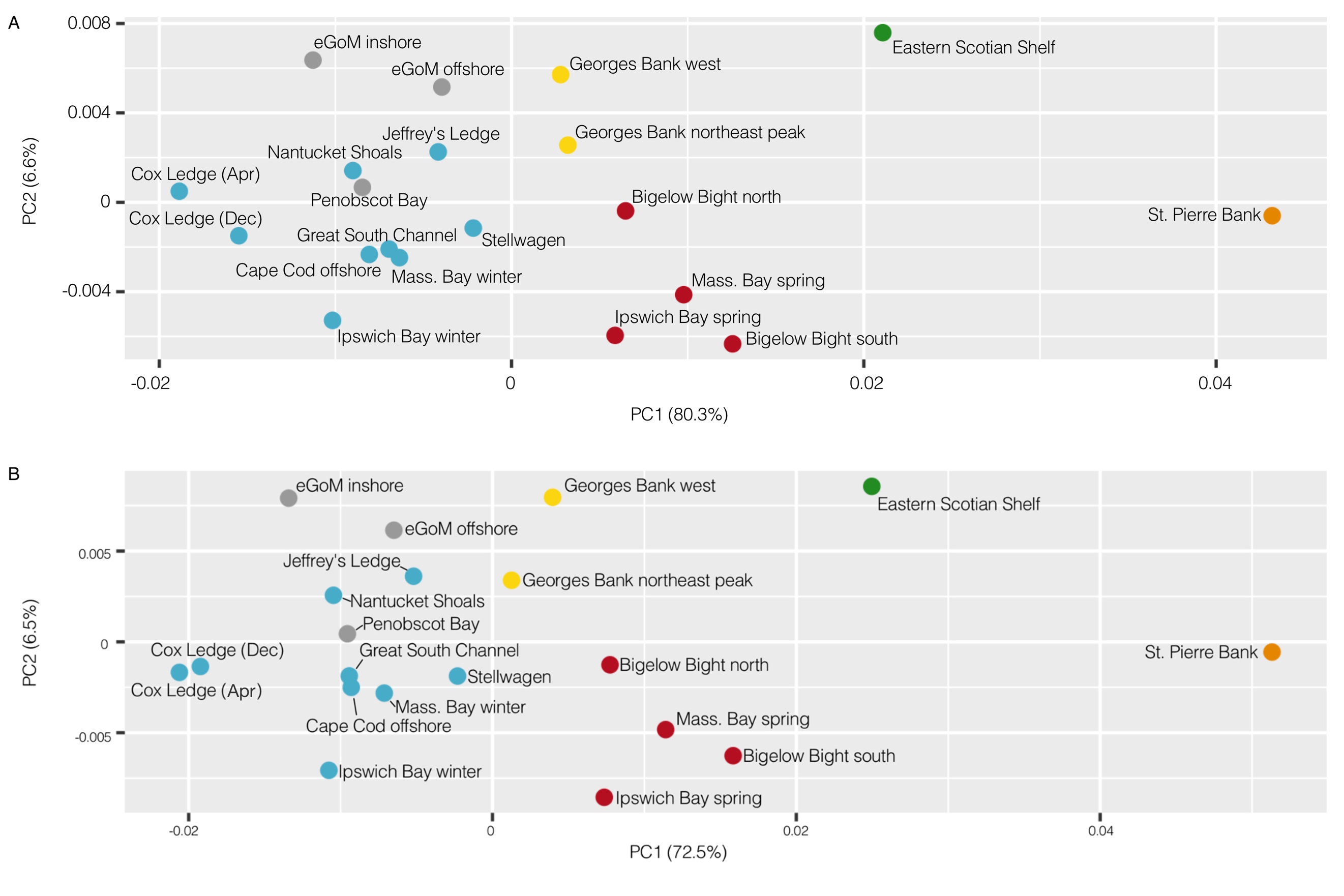


Supplementary Figure 5. MDS plots showing the population structure based on the pairwise F_ST_ matrix calculated with A) the stringent SNP dataset and B) with only eight individuals per sampling location. Points are colored according to our a priori expectations of the population structure as shown in Figure 1 of the main text: red and blue = northern spring coastal complex and southern complex, respectively, of Kovach et al. (2010); yellow = Georges Bank; green = eastern Scotian Shelf; orange = St. Pierre Bank.


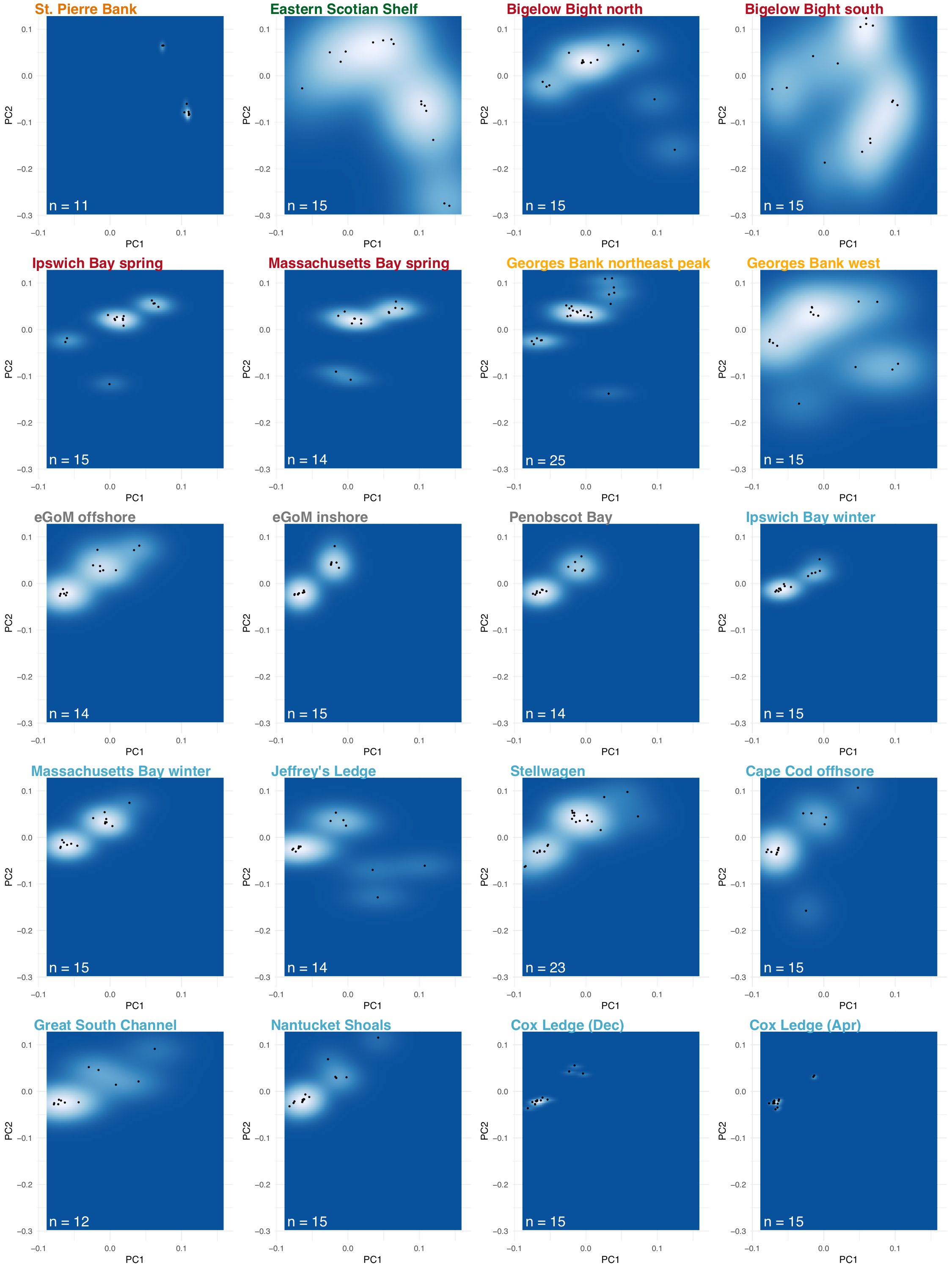


Supplementary Figure 6. Density plots showing the distribution of individuals from each sampling location on principal component axes 1 and 2. The PCA was conducted on all individuals together, but they are plotted separately by sampling location for clarity. PC1 explained 2.71% of the variation while PC2 explained 0.42% of the variation. The labels for the sampling locations are coloured according to our a priori hypotheses of the population structure, as in the main text.


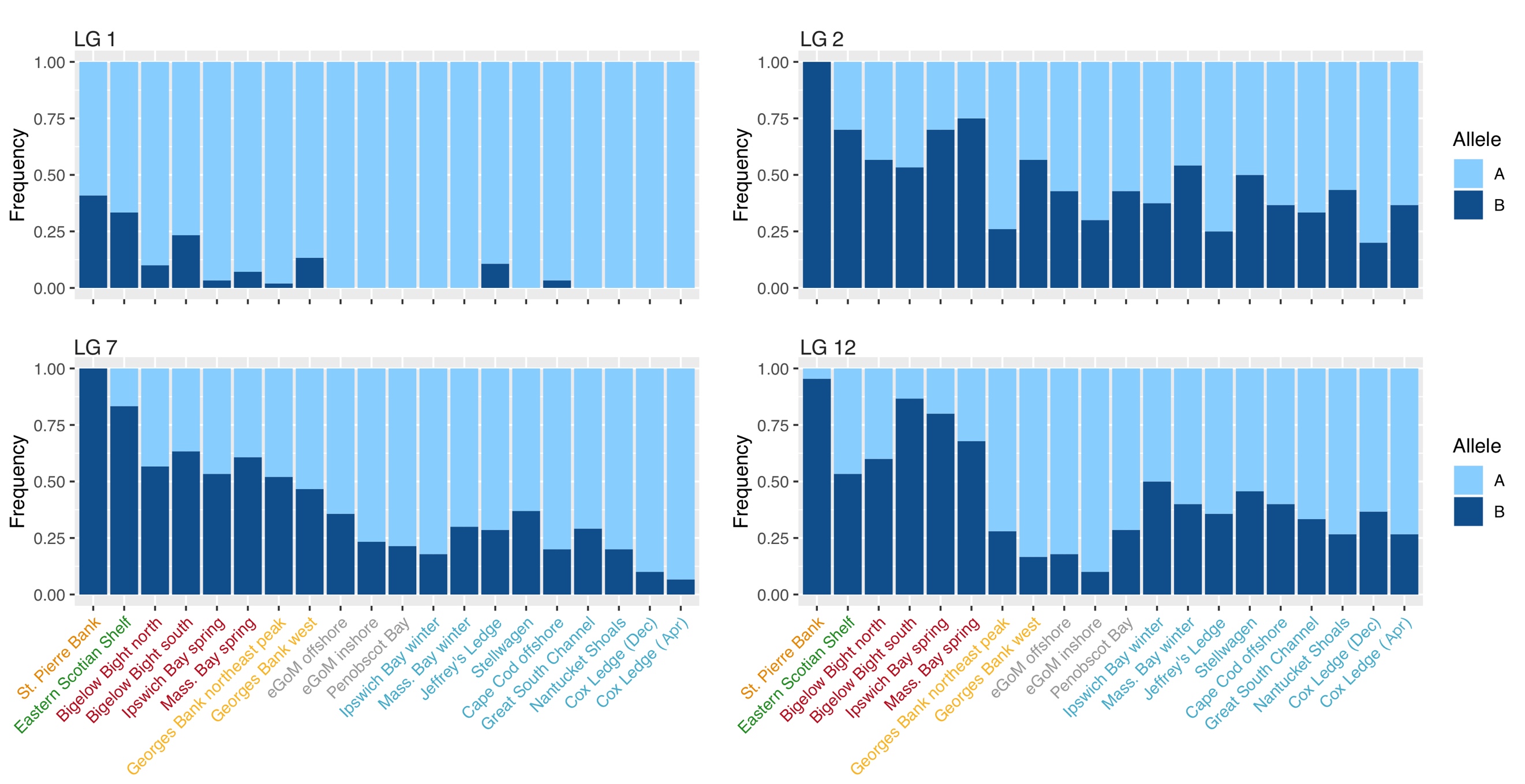


Supplementary Figure 7. Observed haplotype frequencies of the inversions on LG 1, 2, 7, and 12 for each sampling location. The labels for the sampling locations are coloured according to our a priori hypotheses of the population structure, as in the main text.


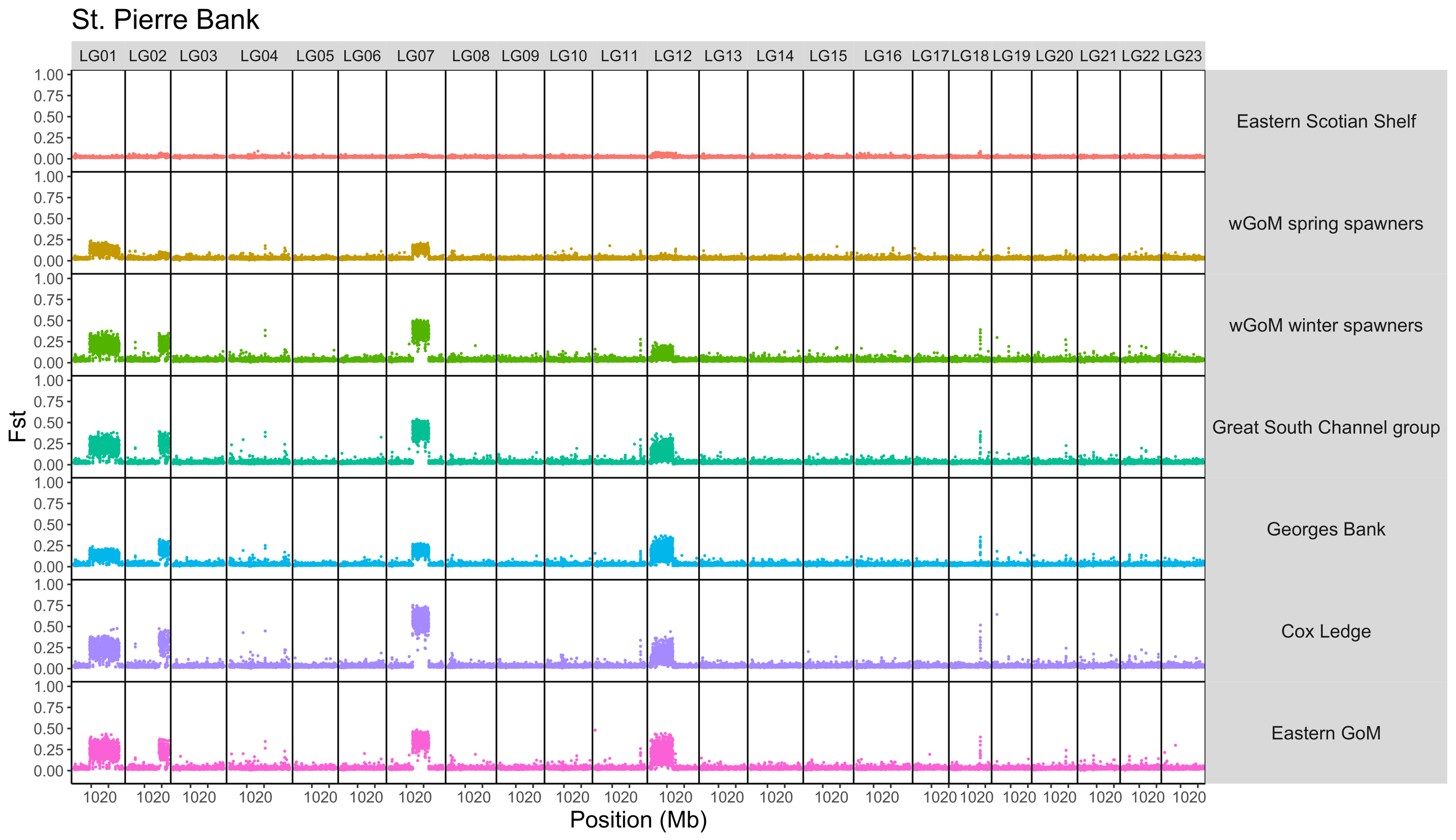


Supplementary Figure 8. Pairwise Manhattan plots of F_ST_ in 15kb windows between St. Pierre Bank and all other groups.


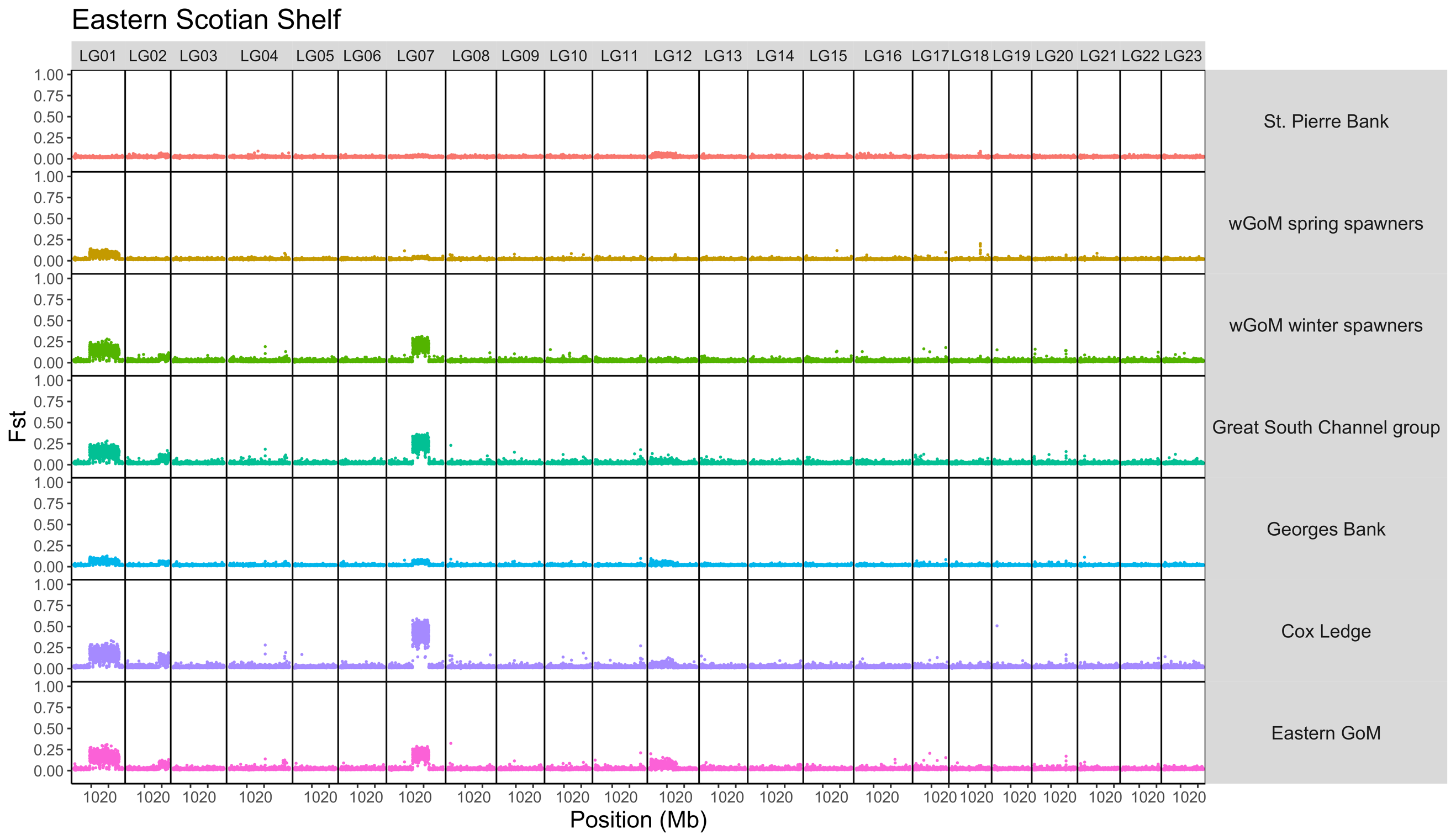


Supplementary Figure 9. Pairwise Manhattan plots of F_ST_ in 15kb windows between the Eastern Scotian Shelf and all other groups.


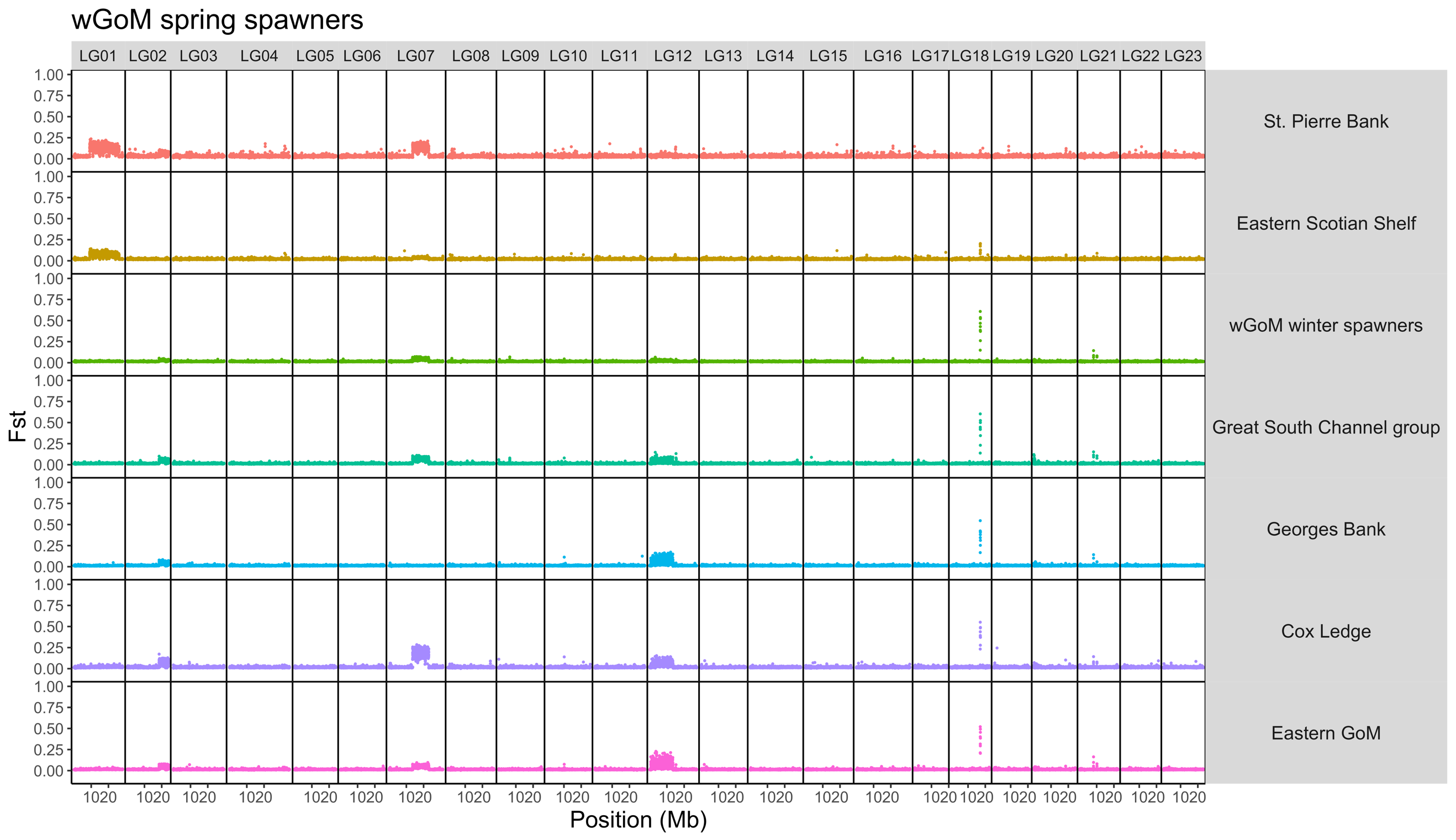


Supplementary Figure 10. Pairwise Manhattan plots of F_ST_ in 15kb windows between the wGoM spring spawners and all other groups.


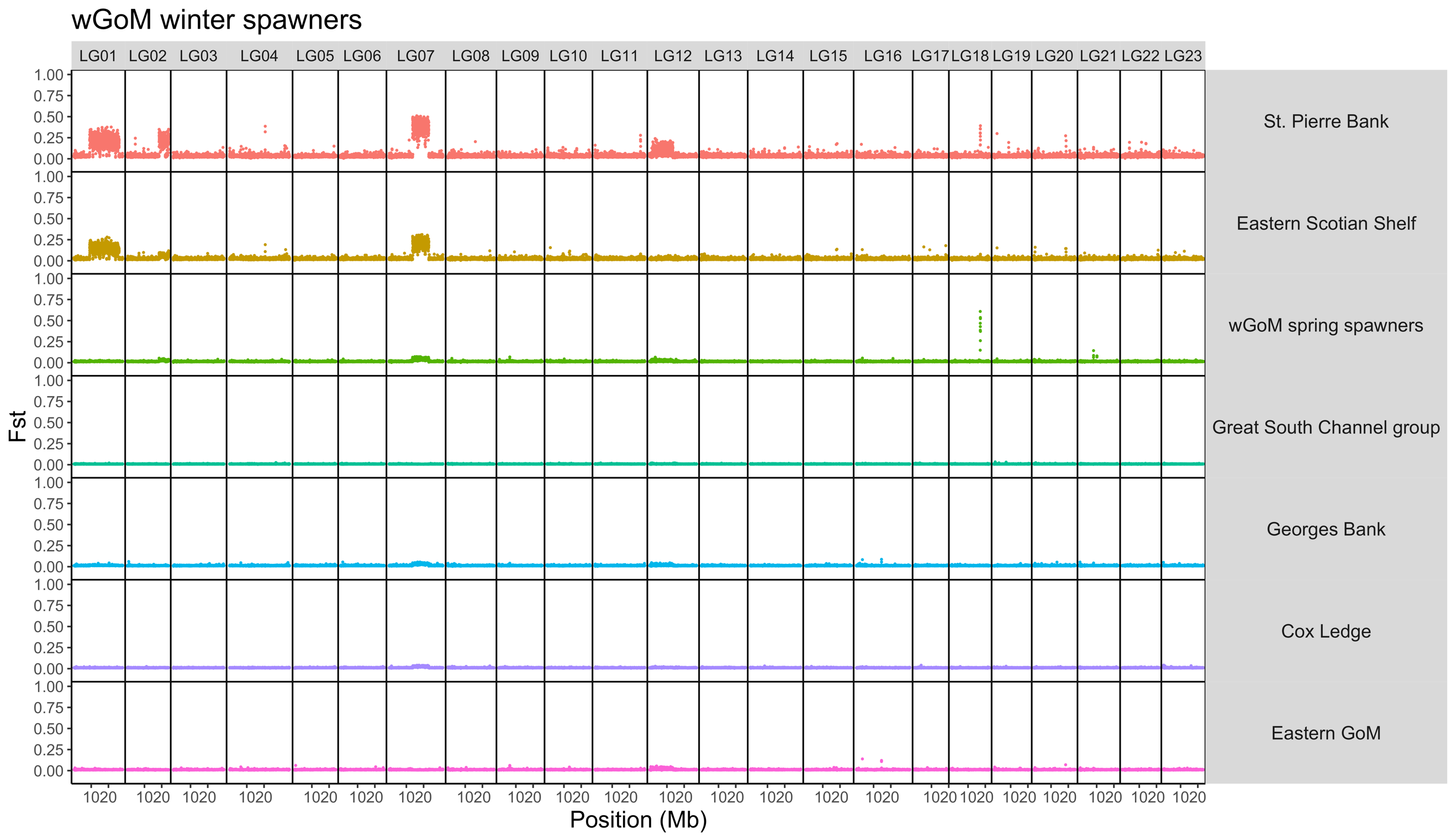


Supplementary Figure 11. Pairwise Manhattan plots of F_ST_ in 15kb windows between the wGoM winter spawners and all other groups.


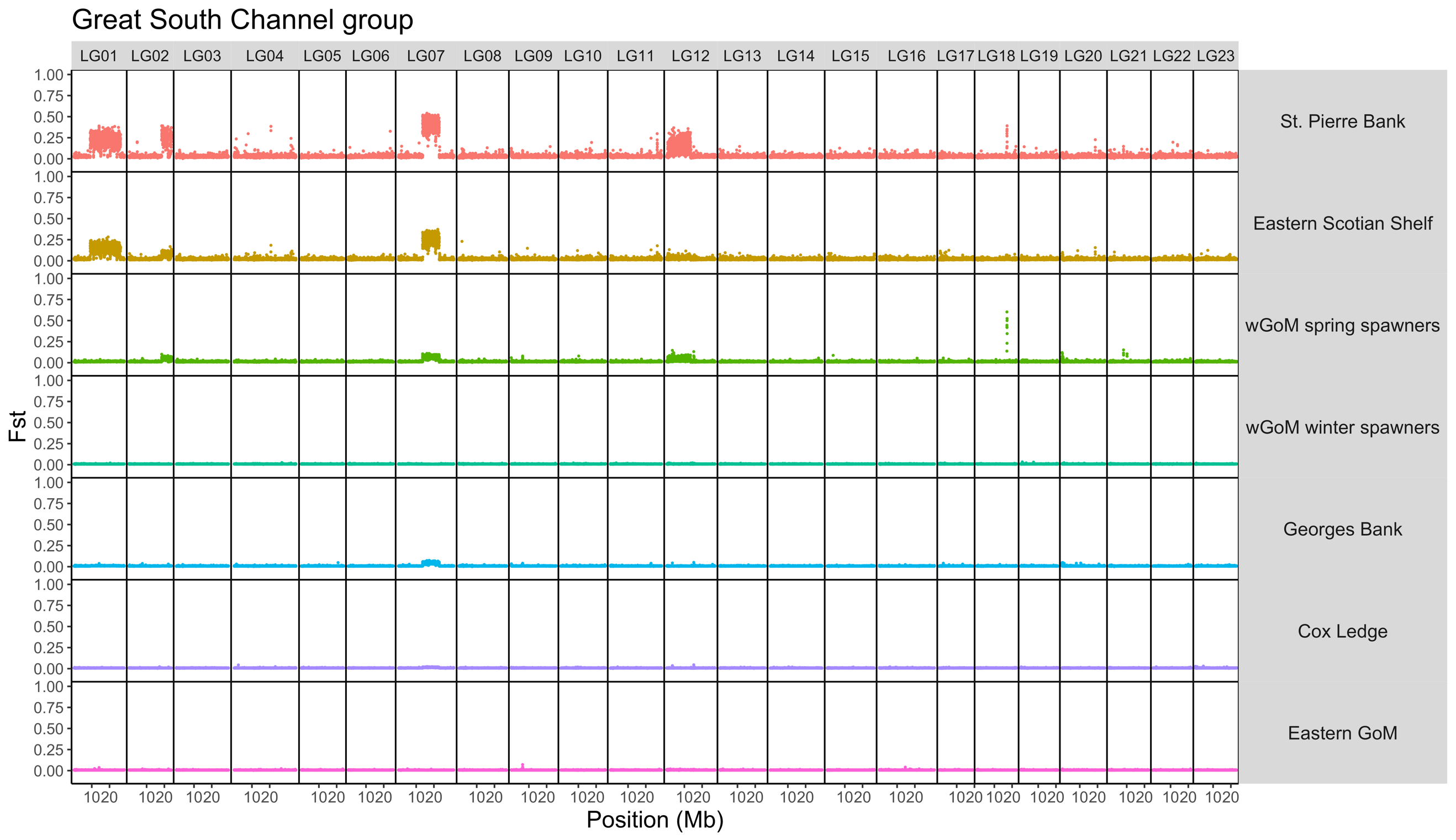


Supplementary Figure 12. Pairwise Manhattan plots of F_ST_ in 15kb windows between the Great South Channel group and all other groups.


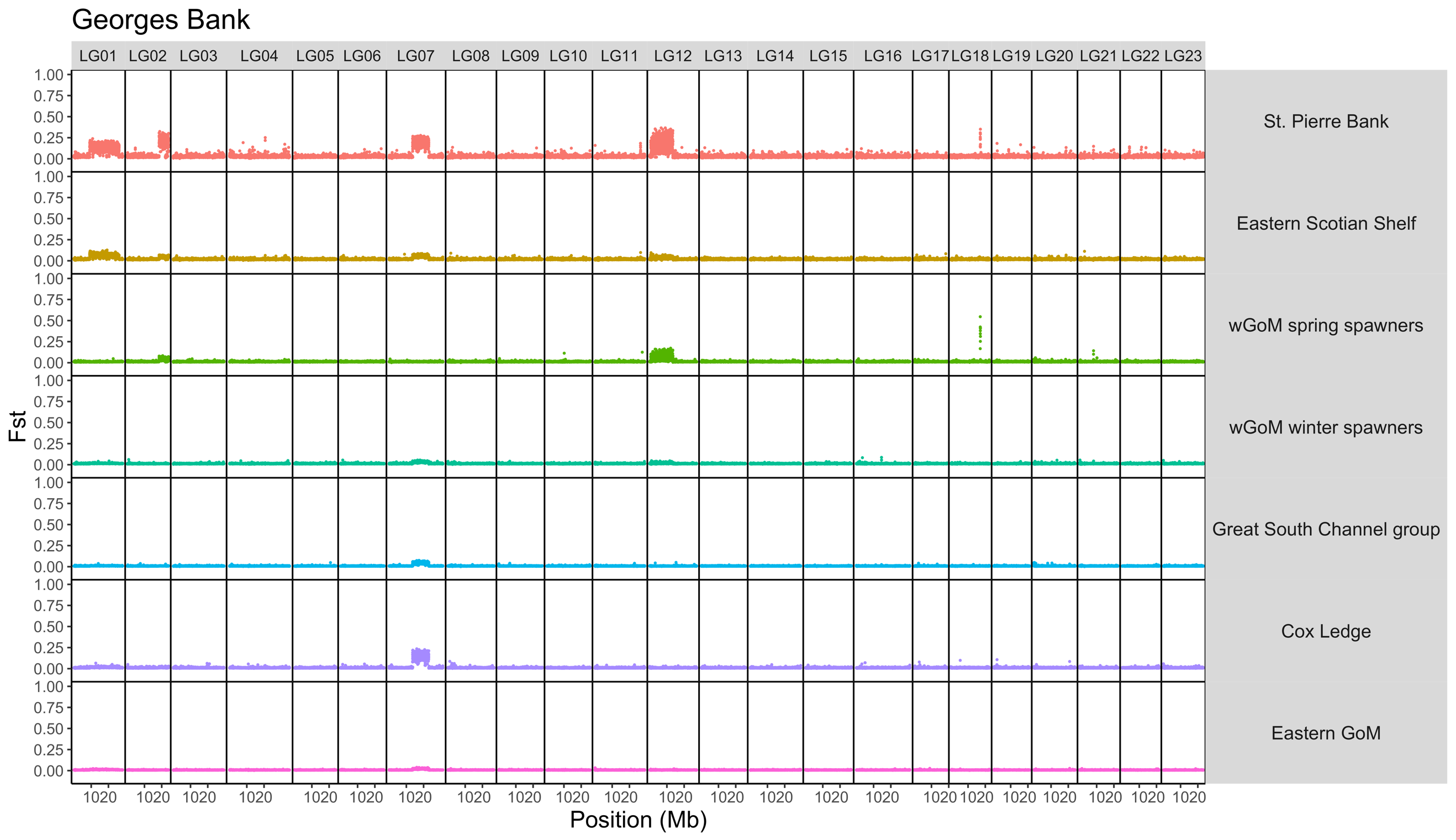


Supplementary Figure 13. Pairwise Manhattan plots of F_ST_ in 15kb windows between Georges Bank and all other groups.


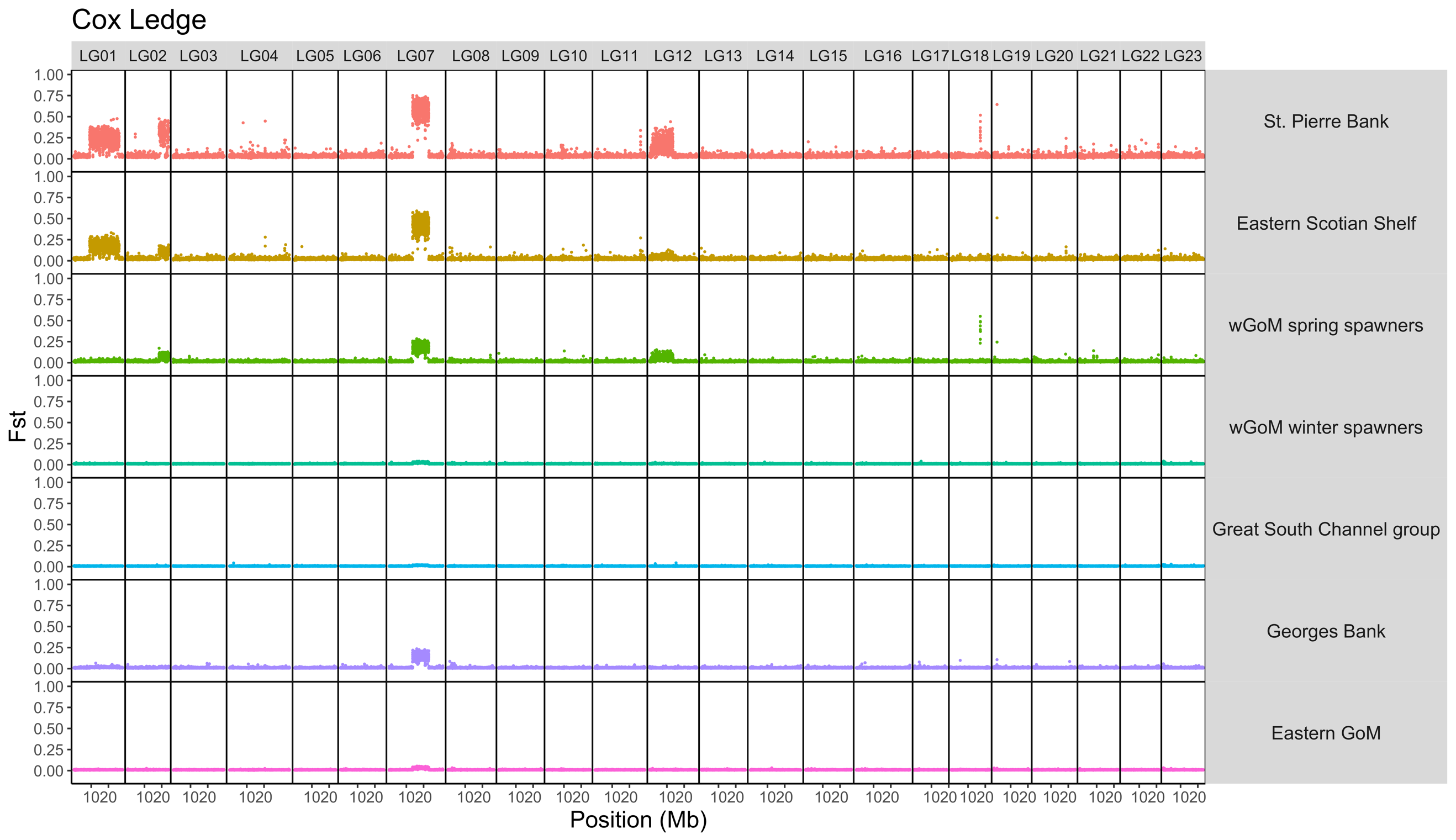


Supplementary Figure 14. Pairwise Manhattan plots of F_ST_ in 15kb windows between Cox Ledge and all other groups.


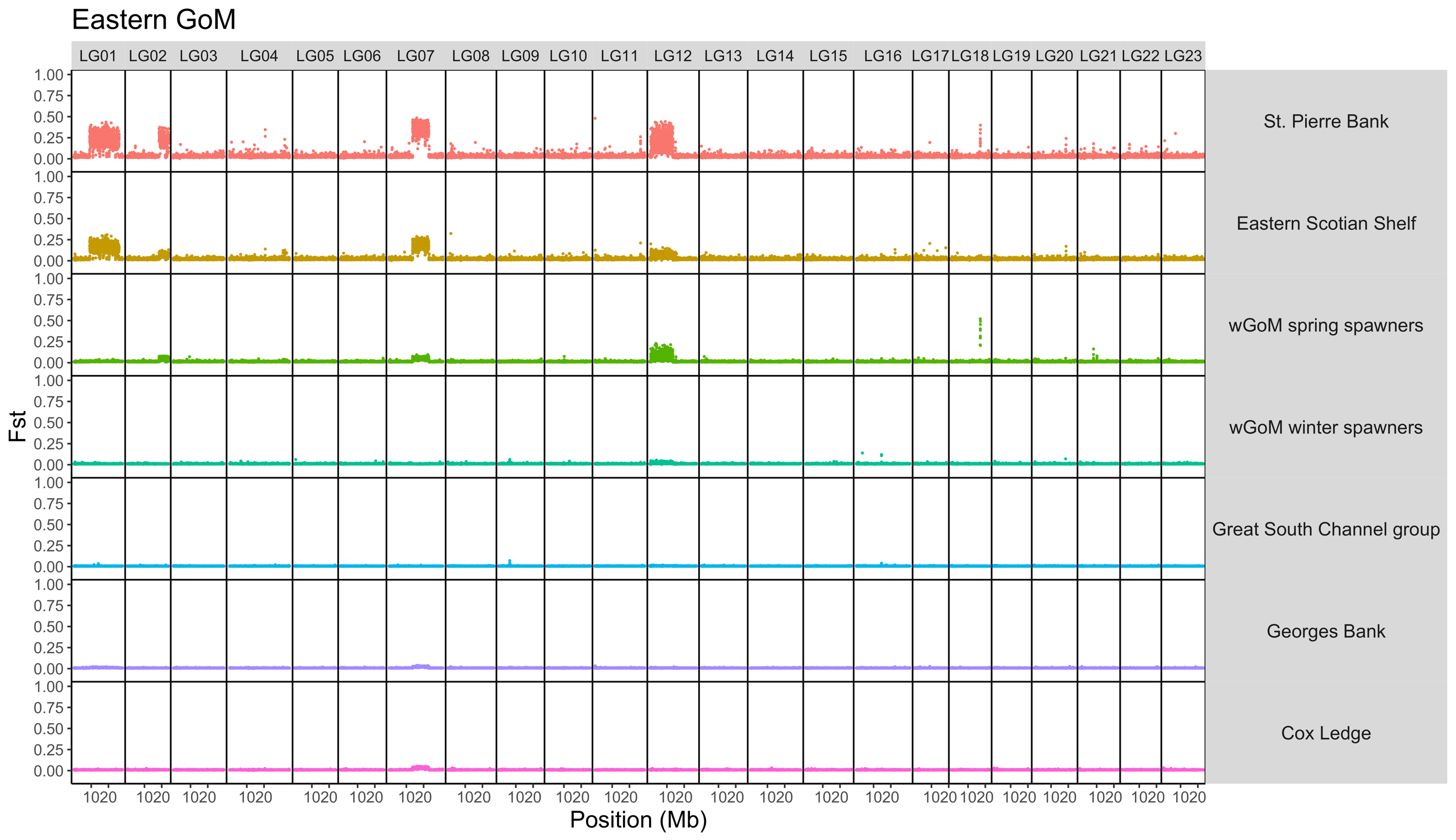


Supplementary Figure 15. Pairwise Manhattan plots of F_ST_ in 15kb windows between Eastern GoM and all other groups.


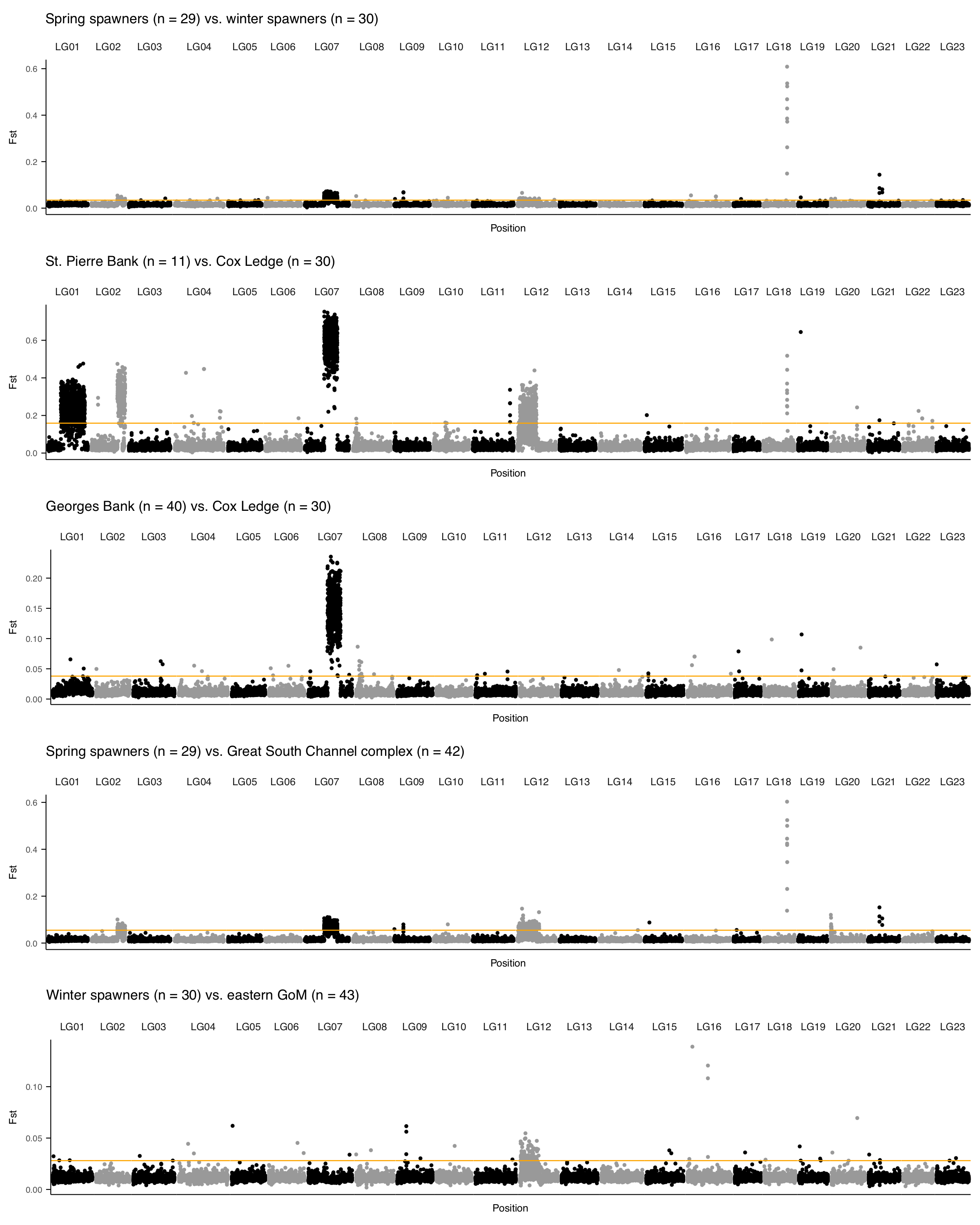
­­­

Supplementary Figure 16. Manhattan plots of F_ST_ in 15kb windows for the five pairwise comparisons that we used to identify outlier peaks. The horizontal orange line in each comparison represents the 99.9^th^ percentile of the windowed F_ST_ distribution when the inversions are excluded. Note, the scale of the y-axis changes among plots. The number of individuals (n) in each group or sampling location is given in parentheses.

Supplementary Figure 17. F_ST_ in 5kb windows and per site D_XY_ estimated between St. Pierre Bank and Cox Ledge for the region on LG08 that overlies the heat shock proteins. The 5kb window on the left that shows FST > 0.3 was not considered an outlier by our criteria, as we based our outlier analysis on 15kb windows of the genome, rather than the 5kb windows displayed here, to reduce some of the variability seen with these smaller windows.


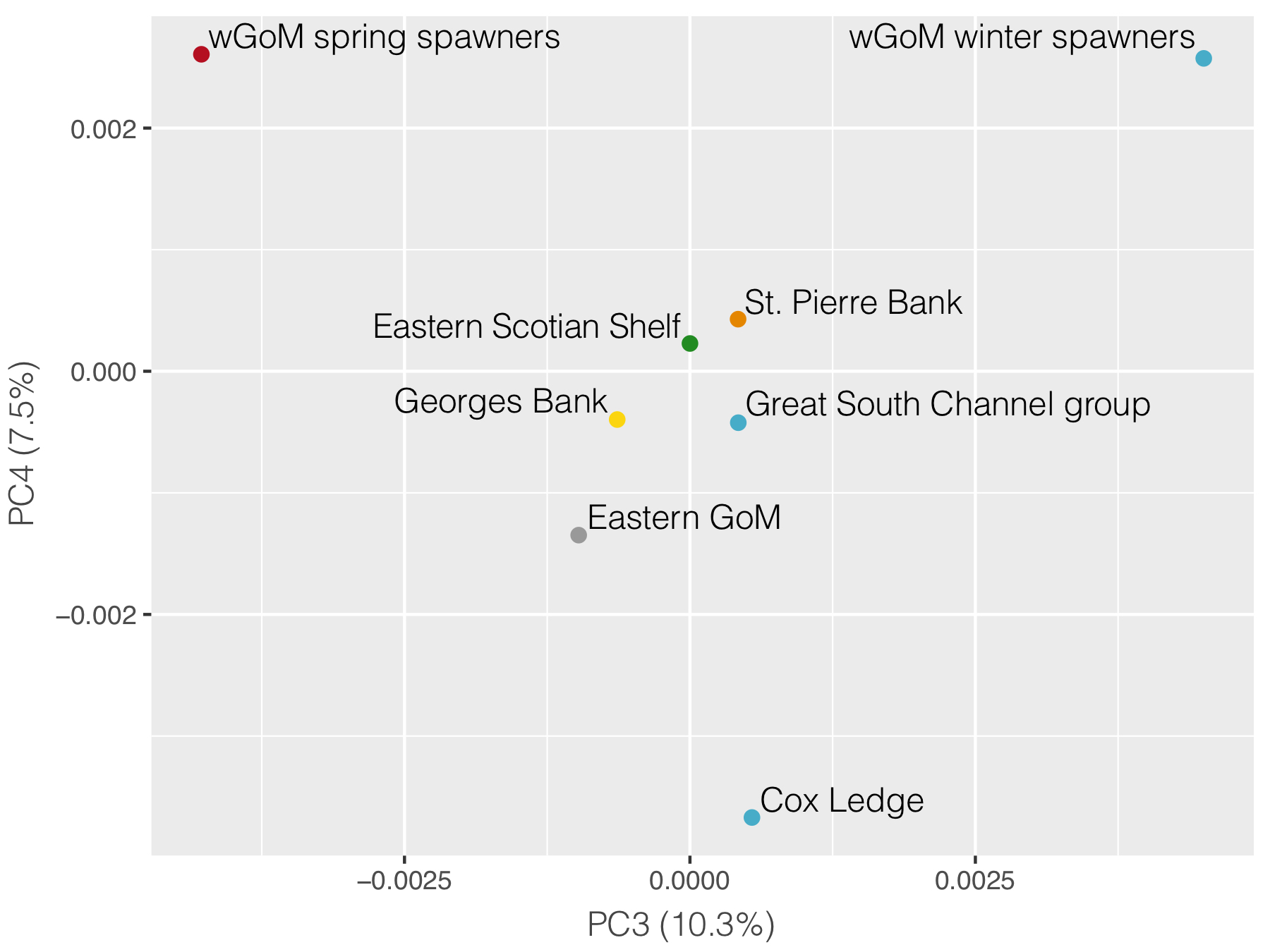
­

Supplementary Figure 18. MDS plot showing the population structure based on the neutral SNP data set for PC3 and PC4. The colours represent our a priori understanding and expectations of the population structure: red and blue = northern spring coastal complex and southern complex, respectively of Kovach et al. (2010); yellow = Georges Bank; green = eastern Scotian Shelf; orange = St. Pierre Bank; grey = eastern GoM.


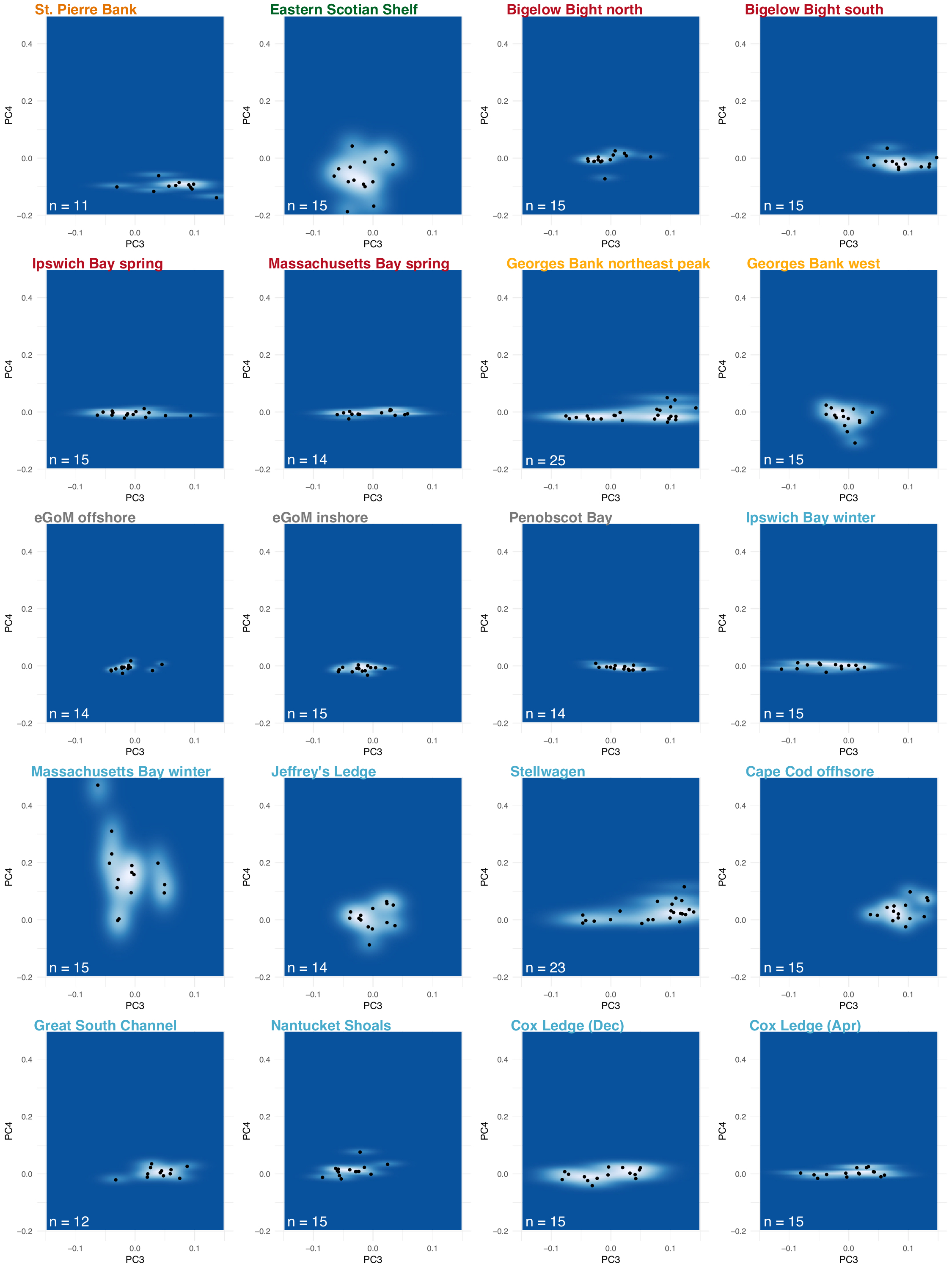


Supplementary Figure 19. Density plots showing the neutral population structure on principal component axes 3 and 4, which explained 0.35% and 0.34% of the variation, respectively. Axes 1 and 2 (not shown) differentiated a couple of individuals from Stellwagen and eastern GoM, respectively, and explained 0.38% and 0.37% of the variation. The PCA was conducted on all individuals together using the neutral SNP dataset; they are plotted separately by sampling location for clarity. The labels for the sampling locations are coloured according to our a priori hypotheses of the population structure, as in the main text and other figures.
